# Actively Learning to Learn Causal Relationships

**DOI:** 10.1007/s42113-023-00195-0

**Published:** 2024-01-05

**Authors:** Chentian Jiang, Christopher G. Lucas

**Affiliations:** School of Informatics, University of Edinburgh, Edinburgh, UK

**Keywords:** Causal learning, Active learning, Transfer learning, Overhypotheses, Generalization

## Abstract

How do people actively learn to learn? That is, how and when do people choose actions that facilitate long-term learning and choosing future actions that are more informative? We explore these questions in the domain of active causal learning. We propose a hierarchical Bayesian model that goes beyond past models by predicting that people pursue information not only about the causal relationship at hand but also about causal overhypotheses—abstract beliefs about causal relationships that span multiple situations and constrain how we learn the specifics in each situation. In two active “blicket detector” experiments with 14 between-subjects manipulations, our model was supported by both qualitative patterns in participant behavior and an individual differences-based model comparison. Our results suggest when there are abstract similarities across active causal learning problems, people readily learn and transfer overhypotheses reflecting these similarities. Moreover, people exploit these overhypotheses to facilitate long-term active learning.

**Supplementary Information:**

The online version contains supplementary material available at 10.1007/s42113-023-00195-0.

## Introduction

A key feature of human cognition is that when we learn, we often acquire knowledge and skills we can use in the future, improving our performance and future learning (e.g., Gick & Holyoak, 1980; Schulz & Gopni, 2004). For instance, cooking one dish can help us learn how to work with ingredients in another dish, practicing one musical instrument can help us learn a new instrument more quickly, and playing with the user interface of one smartphone can also help us learn to navigate another smartphone. In each case, we can learn general patterns and principles that help us set up more specific hypotheses in a new context or problem. For example, in the case of smartphones, we can learn that phones generally make the camera easily accessible. For a new phone, we can then hypothesize that a lock-screen button, quick swipe, or another shortcut will lead to the camera app. Such abstract knowledge that sets up more specific hypotheses in a new context or problem can be called *overhypotheses* (Goodman, 1955; Kemp et al., 2007). Overhypotheses enable us to focus on learning just the novel aspects of future problems and produce a “learning to learn” effect, where we learn more efficiently in the future.

However, it is not well understood how and when we seek out the evidence needed to learn overhypotheses. Do people tend to focus narrowly on learning the task at hand, so that learning overhypotheses happens incidentally? Or do we preferentially choose actions to update our overhypotheses about the abstract nature of families of systems and problems? When we update our overhypotheses in light of new evidence, does that in turn facilitate more informative actions in a new situation? These are questions about how we *actively* learn to learn. To begin answering these questions, we focus on a simple problem domain within active causal learning. We study how people choose actions to probe a sequence of causal systems, where it is critical to learn both the specifics of each system and overhypotheses capturing their similarities.

### Prior Models of Active Causal Learning

While the term active learning can have many meanings, we use it to refer to situations in which learners pick actions to gather their own information (Gureckis & Markant, 2012), as opposed to observing a fixed and given set of information. In particular, we focus on how people seek information that is most helpful given their beliefs and uncertainty, allowing them to discriminate between hypotheses they have in mind. In causal learning, actions or *interventions* can further provide information that is unavailable under observation alone, and this information is critical for discriminating between causal relationships (Pearl, 2009). How adults and children choose interventions, i.e., how they perform active causal learning, has been neatly formalized in computational models (e.g., Steyvers et al., 2003; Cook et al., 2011; Bramley et al., 2015; Coenen et al., 2015; Bramley et al., 2017; Coenen et al., 2019).

**Fig. 1:**
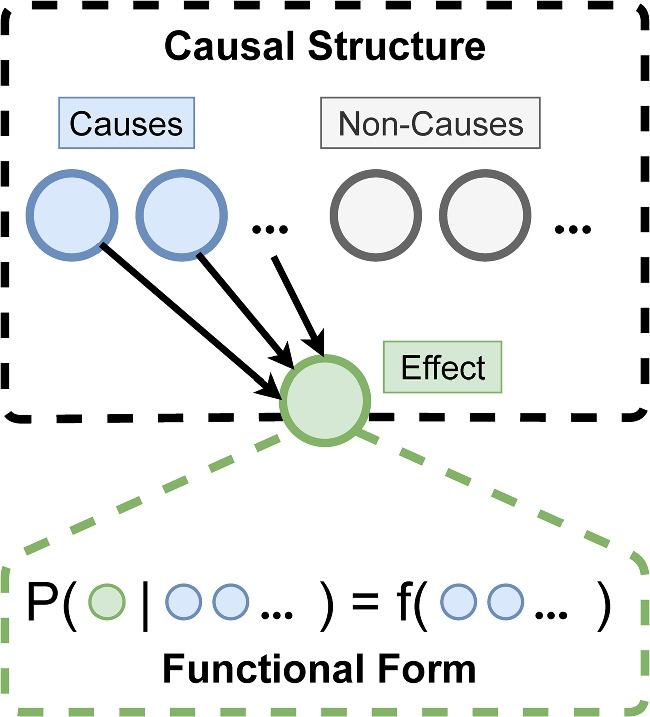
Causal graph. The *causal structure* defines what variables are causes and effects of other variables. The *functional form* is a function of its causes and it defines the conditional probability of the effect given its causes

These past models have focused on how people learn about *causal structure*, which defines what variables are causes and effects of other variables (Fig. 1). Consider an example where a child conducts a small science experiment to test which batteries in their drawer are good or bad. The child devises an intervention strategy based on inserting batteries into a simple circuit with an LED light. The LED is known to illuminate when at least one good battery is in the circuit. Here the child has set up a causal system where the variables are the batteries’ presence in the circuit and the LED’s illumination. Common sense about electrical systems dictates that the LED illumination is the only candidate for being an effect. Therefore, the child is trying to choose interventions to solve the remaining causal structure learning problem of identifying whether batteries are good (causes of the LED’s illumination) or bad (non-causes).

In order to disambiguate between causal structures in a way that is informative from an information-theoretic perspective, interventions should be chosen with the goal of reducing uncertainty about causal structures. This uncertainty reduction is also called information gain (Oaksford & Chater, 1994). Maximizing information gain corresponds to choosing interventions that can quickly narrow down which beliefs are most likely correct. In our LED example, if the child only has two batteries to test, then they are trying to learn which causal structure is correct among four possibilities: neither battery is good, only the first is good, only the second is good, or both are good. Intervening on a single battery would be highly informative because this intervention eliminates half of the possibilities at once: If the LED illuminates, the child can rule out half of the structures where the intervened battery is *not* good, and if the LED does not illuminate, then the child can rule out the other half.

The alternative of intervening on both batteries *can* be highly informative, depending on the child’s prior beliefs. If the child strongly expects a causal structure where neither battery is good, then intervening on both of them would be highly informative because this intervention is expected to result in an *unlit* LED—an outcome that eliminates all three other causal structures at once. However, if the child instead expects at least one battery is good, then intervening on both would no longer be informative: The LED is expected to illuminate, eliminating only the single possibility of neither battery being good.

Past models (e.g., Steyvers et al., 2003; Bramley et al., 2015; Coenen et al., 2015) found that maximizing information gain about causal structures produced good predictions of people’s interventions. However, they made an important simplifying assumption: they only represented people’s beliefs about causal structure within a single, isolated causal learning problem. These models do not predict that people actively “learn to learn”, improving their interventions for learning new causal relationships in the future. In order to formalize such behavior, it is critical to represent *causal overhypotheses*—abstract beliefs about causal relationships that span multiple situations and constrain how we learn the specifics in each situation (Goodman, 1955; Kemp et al., 2007; Lucas et al., 2014; Sim & Xu, 2017). If people’s interventions help them update causal overhypotheses in past learning problems, then in a new problem, they would not need to start from scratch but can choose interventions that are guided by these overhypotheses. In this way, interventions from one situation are able to influence interventions in another, allowing them to adapt and improve not just within the current situation but also across to future ones.

### Overhypotheses About the Functional Form

To accommodate causal overhypotheses, we propose a hierarchical Bayesian model that represents beliefs at multiple levels of abstraction, including both lower-level hypotheses about the current causal relationship and higher-level overhypotheses about the general properties of causal relationships. Like previous rational (Anderson, 1990) models of active learning (e.g., Oaksford & Chater, 1994; Nelson & Movellan, 2000; Steyvers et al., 2009; Bramley et al., 2015; Coenen et al., 2015), we frame causal learning in Bayesian terms and take the active learner’s goal to be finding interventions that maximize information gain, with one key difference: We posit that learners have overhypotheses that they update in light of new evidence, and seek information not just about the causal relationship at hand, but also about these overhypotheses.

We focus on a problem setting involving very simple causal structures and overhypotheses, where it is plausible that people approach our model’s Bayes-optimal behavior and that our model’s predictions will align with approximate models of active causal learning (e.g., Steyvers et al., 2003; Bramley et al., 2017). Here we can provide a first account of overhypothesis transfer in active causal learning that focuses on people’s high-level *goals*: Do they pick interventions for the purpose of learning about overhypotheses, which can facilitate long-term learning? How do they trade this off with their short-term learning goals? We, however, do not argue that people choose Bayes-optimal interventions in general and for arbitrarily complex problems: Past work suggests that people, faced with complex problems, perform resource-constrained and approximate reasoning by sampling (Denison et al., 2013; Bonawitz et al., 2014; Zhu et al., 2020; Sanborn et al., 2021) or considering only a few hypotheses at a time (Steyvers et al., 2003; Bramley et al., 2017). Modeling these resource-constrained and approximate processes would improve our understanding of how people realize their overhypothesis learning goals in more challenging domains, and we believe our work sets the stage for building such process-level models in the future.

While our general approach can be applied to arbitrary overhypotheses, we focus here on overhypotheses about the *functional form* of causal relationships. The functional form governs how causes combine or interact to produce an effect (Fig. 1), e.g., are multiple causes necessary to bring about an effect? Do relationships tend to be deterministic or stochastic? Going back to our LED example, if we assume that good batteries (causes) are interchangeable, then we can express the functional form in terms of the voltage threshold for illuminating the LED (effect), or—in terms of our original variables—the number of good batteries that are required to make it illuminate. The functional form can also capture how reliable we expect the illumination to be, and how that varies with the number of causes; the illumination might be deterministic if our voltage threshold is exceeded by any amount, or it might be noisy if our threshold is barely exceeded.

We focus on beliefs about the functional form as a type of overhypotheses because we can build on simple hierarchical models that include functional forms and give good accounts of human learning in the absence of an active learning element (Lucas & Griffiths, 2010). Although there are other types of overhypotheses for causal learning, the functional form is, to our knowledge, the most commonly studied kind and is representative of the causal overhypothesis literature (Griffiths & Tenenbaum, 2009; Lucas & Griffiths, 2010; Lucas et al., 2014; Kosoy et al., 2022; Lu et al., 2016). Additionally, varying overhypotheses in this setting leads to clear and systematic differences in what interventions are more informative. In contrast, we would expect subtler effects in an experiment based on other salient studies of overhypotheses (e.g., Kemp et al., 2007; Austerweil et al., 2019), owing to fewer degrees of freedom in possible interventions and the possibility of greater individual variability in prior beliefs.

To understand how overhypotheses about functional forms differ from normal hypotheses, we refer to Kemp et al. ’s (2007) definition of overhypotheses: “any form of abstract knowledge that sets up a hypothesis space at a less abstract level”. Now consider beliefs that favor functional forms where two or more causes are needed to produce an effect. In our LED example, this would mean two or more good batteries are needed to light an LED.^1^ We can “[set] up a hypothesis space at a less abstract level” by considering how these beliefs set up more specific form-structure combinations that are likely to govern a particular set of batteries and LED. For example, likely hypotheses could be *there is a set of batteries that can light up the LED in pairs*, or *there is a single good battery that is not detectable with the LED*. While both causal structure and functional form knowledge are needed to set up these situation-specific hypotheses, we focus on a setting where they are not on equal footing: The structure is only about the situation-specific set of causal variables (e.g., a particular set of batteries and LED), but beliefs about the form are also “abstract knowledge” that can be abstracted, or lifted, out of one situation and reused for learning in future situations with novel causal variables (e.g., use the *two or more* belief as a starting point to understand novel batteries and LEDs). Thus, whereas the causal structure is a hypothesis, beliefs about plausible functional forms across different circuits are overhypotheses.

**Fig. 2:**
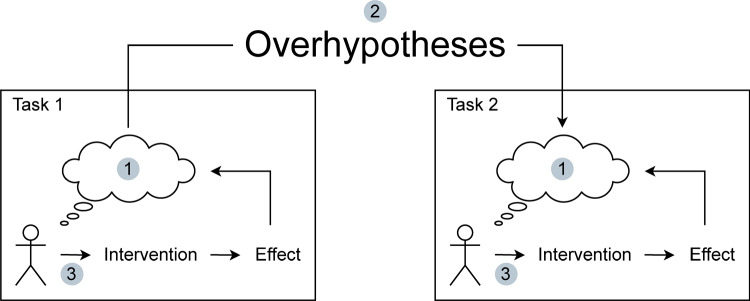
Hierarchical Bayesian model. Each circled number represents one of the three scientific hypotheses of our model: (1) people represent rich overhypotheses; (2) people transfer and adapt their overhypotheses from one task (left box) to the next (right box); and (3) they sacrifice short-term learning for information gain about overhypotheses

To illustrate how overhypotheses about the functional form can affect future intervention choices, now suppose that we are in a new situation with new causal variables and we do not know the functional form. Here we can transfer and rely on overhypotheses that we have previously learned in similar situations. As we have seen throughout the LED example, we might have acquired overhypotheses that favor *disjunctive* functional forms, where only a single cause is necessary to produce an effect, or overhypotheses that favor *conjunctive* functional forms, where we need two or more causes. Under disjunctive-favoring overhypotheses, intervening on a single variable at a time would be informative about which variables are causes of an effect, e.g., testing singleton batteries would reveal a good battery whenever there is an LED illumination. However, under conjunctive-favoring overhypotheses, this strategy would not be informative at all. Conjunctive overhypotheses expect that no single variable, cause or non-cause, is sufficient to produce an effect. For example, intervening on a single battery would always result in an unlit LED. This outcome provides no information about whether that battery is good (but just not sufficient by itself) or bad. Instead, having conjunctive overhypotheses leads us to a different strategy of testing two or more batteries at a time. Now it is possible to cause LED illuminations that tell us our intervention contains at least two good batteries.

Since past models of active causal learning have largely focused on learning causal structure (e.g., Bramley et al., 2015; Coenen et al., 2015; Steyvers et al., 2003), they have tended to assume the functional form was known in advance to experimental participants, or that the functional form was consistent with the simple expectation that a single cause was sufficient to produce or prevent an effect. This assumption of causal sufficiency holds for a wide variety of phenomena in causal inference and appears to be a default expectation people have in unfamiliar contexts (Cheng, 1997; Gopnik & Sobel, 2000; Tenenbaum & Griffiths, 2001; Griffiths & Tenenbaum, 2005; Griffiths et al., 2011; Lu et al., 2008), but it is not always appropriate. Both children and adults can adjust their overhypotheses to learn other functional forms, where multiple causes may be needed to produce the effect (e.g., the conjunctive form), and they are able to transfer these overhypotheses to guide their causal inferences in new tasks (Lucas & Griffiths, 2010; Lucas et al., 2014; Kosoy et al., 2022; Griffiths & Tenenbaum, 2009; Lu et al., 2016). In our hierarchical Bayesian model, we accommodate uncertainty in peoples’ overhypotheses about the functional form. In situations where people might be expected to have very strong prior expectations about the functional form, our model is essentially equivalent to Steyvers et al. ’s (2003) Rational Identification model and Bramley et al. ’s (2015) Scholar model. In other situations where the form is not known in advance and when many forms are possible, our model makes substantially different predictions.

### Our Hypotheses

Through our hierarchical Bayesian model, we formalize three scientific hypotheses (Fig. 2): (1) People represent rich overhypotheses; (2) people transfer and adapt their overhypotheses across tasks; and (3) they sacrifice short-term learning for information gain about overhypotheses. Below, we discuss the modeling contribution and behavioral insights of each hypothesis. We also provide an overview of how we test each hypothesis by comparing our model with ablations of our model, which remove one hypothesis at a time (see “A Model of Actively Learning to Learn” for implementation details).

#### Hypothesis 1: People Represent Rich Overhypotheses

First, our model posits that people represent rich overhypotheses that can accommodate a large variety of functional forms. Our model uses an extended version of Lucas and Griffiths ’s (2010) *sigmoid* space of functional forms. The sigmoid space is computationally simple but is able to express variations in (1) the number of causes required to generate an effect and (2) the reliability of the effect. The common disjunctive (1 cause; deterministic effect) and conjunctive (2 causes; deterministic effect) forms are neatly contained as special cases.

##### Modeling Contribution

Our model’s space contains 400 variations of sigmoid forms and is richer than spaces used in previous works: Compared with past models of active causal learning (e.g., Bramley et al., 2015; Coenen et al., 2015; Steyvers et al., 2003), our model removes the assumption of having only a single functional form and predicts that interventions discriminate between many possible forms. Compared with past models and studies of causal overhypotheses (Griffiths & Tenenbaum, 2009; Lucas & Griffiths, 2010; Lucas et al., 2014; Lu et al., 2016; Kosoy et al., 2022), our work goes beyond studying 0–2 causes as the threshold for generating an effect. Our model and Experiment 2 introduce new forms where the effect requires at least 3 causes (under varying degrees of noise)—we call this “3-conjunctive”. This addition enables us to study a wider coverage of generative and noisy overhypotheses. Moreover, in Experiment 2, not only can we study transfer to tasks requiring qualitatively different intervention strategies (testing singletons for disjunctive tasks vs. multiple objects for conjunctive tasks), but also transfer to tasks requiring *similar* strategies (testing multiple objects for both 3-conjunctive and conjunctive tasks).

##### Behavioral Insight

It is not obvious that people track or represent multiple possibilities in our model’s space of functional forms at once, much less choose interventions that efficiently discriminate between plausible possibilities. Rather, a common alternative from the active learning literature is the *positive testing strategy* (Klayman & Ha, 1987; Nickerson, 1998; Wason, 1960). Here interventions would only try to produce positive evidence (i.e., the “on”, but not “off”, effect) as a way to confirm a single functional form. The rest of our proposed space of various generative and noisy forms would be ignored. Similarly, Steyvers et al. ’s (2003) work suggests people choose interventions that focus more on testing individual causal hypotheses (they were well-explained by “Rational Test” models) than on discriminating between hypotheses within a large space of possibilities.

In our setting, positive testing can manifest as people trying to confirm their prior preferences for a particular disjunctive and deterministic form (Lucas and Griffiths , 2010; Lu et al. , 2008; Mayrhofer and Waldmann , 2016; Schulz and Sommerville , 2006): Their interventions may test one object at a time, anticipating that a single object would be sufficient and reliable for producing the effect. Such interventions would not consider alternative overhypotheses like conjunctive-favoring ones, where at least two causes are needed to produce the effect and so are only revealed by testing combinations of objects. Moreover, we expect positive testing to be more likely among participants who wish to expend less cognitive effort, as is supported by Coenen et al. ’s (2015) work in a similar causal learning setting (without overhypotheses). There may be several such participants who are less motivated to perform well in our experiments, but instead want to complete the experiments quickly and receive their performance-independent compensation.

To test our model’s sigmoid space, we compare our full model against a “Fixed-Form” ablation model. This ablation model is essentially equivalent to past active causal learning models, such as Steyvers et al. ’s (2003) Rational Identification Model and Bramley et al. ’s (2015) Scholar Model; it reduces the space of functional forms to a single deterministic and disjunctive form to represent the past assumption that a single cause is reliable and sufficient for producing an effect.

#### Hypothesis 2: People Transfer and Adapt Their Overhypotheses

The second scientific hypothesis represented by our model is that people transfer and adapt their overhypotheses. By this, we do not mean that people simply continue to hold onto the same overhypotheses across tasks, but that they *adapt* their overhypotheses and interventions across all tasks. Overhypotheses acquired from past tasks would serve as a starting point for further change and learning in a new task. We expect this “learning to learn” behavior to appear in our setting, where tasks are different in a systematic way so that transferring overhypotheses gives opportunities for more efficient learning in new tasks.

##### Modeling Contribution

Past models have either accommodated overhypothesis transfer (e.g., Griffiths & Tenenbaum, 2009; Lucas & Griffiths, 2010; Lucas et al.,^2014^; Lu et al., 2016) or active learning (e.g., Bramley et al., 2015; Coenen et al., 2015; Steyvers et al., 2003), but typically not both. By implementing both, our model can generate new kinds of predictions that are not anticipated by earlier models: People can continually adapt their interventions across related tasks by (1) transferring their overhypotheses and (2) seeking information about overhypotheses. Our model is also grounded in information theory and contributes a principled way of testing these predictions. For ease of understanding, we split the intertwined concepts in the predictions, focusing on (1) transfer here and (2) information gain in “Hypothesis 3: PeopleSacrifice Short-term Learning for Information Gain AboutOverhypotheses”.

Importantly, information-theoretic models of active causal learning represent people’s belief adaptation in a particular task by tracking a probability distribution and using Bayesian inference to update this distribution (e.g., Steyvers et al., 2003; Bramley et al., 2015; Coenen et al., 2015). Following from this Bayesian framework, our model carries over a distribution across tasks to represent the transfer of overhypotheses: This distribution is updated using Bayesian inference on evidence from past tasks, is used as a prior in a new task, and continues to be updated in the new task. We use our model to test for transfer behavior in a participant by updating a distribution, which represents their overhypotheses, using the same interventions and observations that were produced by that participant. Transfer is marked by their interventions being informative under a distribution that incorporates evidence from past tasks, as opposed to evidence from only the current task. This test is sensitive to each individual’s unique history of interventions and observations and thus can be used to detect transfer no matter what their past experiences and intervention strategies are. We are not aware of alternative approaches to detecting transfer in this general-purpose way.

To illustrate the value of our model-based analysis, consider the alternative of using a simple statistical metric that counts the number of objects in an intervention. This metric can indicate whether people transferred disjunctive (1 object) or conjunctive (multiple objects) overhypotheses and is used for this purpose in our Experiment 1. However, this metric is limited when we investigate differences in overhypotheses resulting from subtle differences in training tasks or individuals having different intervention strategies. For example, in Experiment 2, transferring overhypotheses from either a conjunctive or 3-conjunctive training task is likely to lead to multiple-object interventions and it is not clear what number of objects would be more indicative of one or the other. And even if people had the same training tasks, their propensity for transfer cannot always be captured with similar values on a simple metric: Consider the situation where one person employed a lazy intervention strategy that did not reveal much about the training task and another person employed a very efficient strategy. We would expect their overhypotheses to be very different (especially if their overhypotheses can have the rich variations proposed by our first scientific hypothesis) and their transfer behavior to look very different even though they had the same training opportunity. Thus, as described above, we use our Bayesian model’s capability to track a distribution per individual to capture transfer in a way that accounts for fine-grained variations in individuals’ experiences and strategies.

##### Behavioral Insight

While there is evidence that people can transfer beliefs about functional forms (Lucas & Griffiths, 2010; Lucas et al., 2014; Griffiths & Tenenbaum, 2009; Lu et al., 2016; Kosoy et al., 2022), this effect could be weak in our setting. We draw from multiple perspectives that show mixed evidence for transfer even when the training and transfer tasks are similar. First, we study adult participants, and past studies have shown that adults, in contrast to children, have a weak transfer effect for conjunctive overhypotheses (Lucas & Griffiths, 2010; Lucas et al., 2014). Second, in contrast to most studies of functional form transfer, we ask participants to produce their own evidence instead of receiving it from the experimenter. While people make strong inferences from examples produced by a teacher or expert—such as the experimenter—they likely make weaker inferences based on their own actions, which are chosen under uncertainty (Shafto & Goodman, 2008). Thus, they may have less confidence in their learning and be less willing to transfer their beliefs to future tasks. Third, we study overhypotheses that are beliefs about functions, and past function learning works have shown that people tend to neglect to transfer (i.e., extrapolate) complex functions; they often fall back to a linear relationship that is consistent with their priors (Kalish et al., 2004; Kalish, 2013). Overall, it is not apparent whether we will find a notable transfer effect in our setting.

One way that our participants may not exhibit transfer behavior is by reverting back to their priors, similar to the function learning setting above. They may have strong priors, specifically ones that favor deterministic and disjunctive overhypotheses (Lucas & Griffiths, 2010; Lu et al., 2008; Mayrhofer & Waldmann, 2016; Schulz & Sommerville, 2006), and treat any learning about alternative overhypotheses as rare. They may then think these alternative overhypotheses are unlikely to be useful again, so instead of transferring these overhypotheses, they rely on the same deterministic- and disjunctive-favoring prior in a new situation. Such behavior is consistent with Zhao et al. ’s (2022) model (LoCaLa) of how people transfer beliefs about causal functions, which are much like our work’s functional forms.

Given the above discussion, it is possible that most people do not transfer their overhypotheses. We implement this possibility as the “No-Transfer” ablation model: Following the above idea that people may revert to their priors, “No-Transfer” predicts people start anew in each task using the same prior, regardless of any learning in previous tasks. We nevertheless hypothesize that our full model, with its transfer idea, will be the dominant predictor of participant behavior. We test our full model by comparing it to “No-Transfer”.

#### Hypothesis 3: People Sacrifice Short-term Learning for Information Gain About Overhypotheses

The final hypothesis our model can be used to test is that people sacrifice short-term learning for information gain about overhypotheses. This hypothesis focuses on the learning opportunities that people create for themselves when they are in control of their learning process: To what extent do people prioritize interventions that are informative for learning overhypotheses, i.e., discriminating between possibilities within a rich space of functional forms (Hypothesis 1)? Would they pursue such overhypothesis learning, which can help them in the future (Hypothesis 2), even when that means sacrificing information gain about their immediate situation, i.e., about causal structures?

##### Modeling Contribution

To quantify the extent that people’s intervention choices are informative for overhypothesis versus causal structure learning, our model first takes an established information-theoretic approach to formalizing the notion of “informativeness”. Given an intervention and its potential outcomes, our Bayesian model can compute their implications for posterior distributions over functional forms and causal structures. The informativeness of an intervention can be formalized in terms of how much it is expected to reduce uncertainty in the posterior distribution. This is commonly referred to as *expected information gain* and is a principled metric from information theory and the broader active learning literature (e.g., Oaksford & Chater, 1994; Nelson & Movellan, 2000).

Like past models of active causal learning (e.g., Bramley et al., 2015; Coenen et al., 2015; Steyvers et al., 2003), our model maximizes expected information gain about causal structures to predict that people seek information about the causal structure at hand. A key difference of our model is that it also maximizes expected information gain about the space of functional forms. Our model can thus predict that people also seek information about overhypotheses, which can then shape their future interventions and inferences.

Moreover, to our knowledge, our model is the first to capture the idea that people can strike a balance between overhypothesis and causal structure learning. Our model performs a novel decomposition of expected information gain about overhypotheses versus causal structures, and balances them with a weight parameter. Although our full model can accommodate any weight values between 0 and 1, it commits to nonzero weights; this implies that a learner attaches some implicit value to overhypothesis learning, even when entails sacrificing opportunities to learn about the causal structure at hand.

##### Behavioral Insights

Previous work has discussed how people gather information that can be useful for the long term: Children’s playful actions seem inefficient at first, but may help them develop cognitive abilities for their adulthood (e.g., Buchsbaum et al., 2012; Chu & Schulz, 2023). And adult scientists can design an experiment with multiple blocks or even an ensemble of multiple experiments, which not only addresses a specific research question but also develops a broader theory that can be applied to future settings (e.g., Ivanova et al., 2021; Almaatouq et al., 2022; Valentin et al., 2023). However, we are not aware of any empirical studies that have contrasted this long-term information-seeking behavior with short-term learning.

We begin to provide insights into people’s preferences for long- versus short-term information gain through our behavioral experiments: Within each individual task, participants are asked to discover the particular causal structure of the task, i.e., identify which variables are causes of an effect. Although their success also depends on understanding the functional form, participants may succeed faster by prioritizing information gain about causal structures and learning about functional forms as a side effect. However, across multiple tasks, participants can benefit from seeking information about functional forms, picking interventions that not only confirm appropriate overhypotheses but also reveal contradictory evidence for adapting and improving their overhypotheses. In the short run, this behavior may delay them from identifying the true causal variables. In the long run, however, they may be able to exploit their overhypotheses in several tasks that share similar functional forms and speed up their learning. Our experiments thus create a setting where we can study how people balance overhypothesis learning with short-term learning about causal structures.

Our model makes the prediction that people sacrifice causal structure learning for information gain about overhypotheses. To test this prediction, we evaluate how well our model predicts participant interventions in our experiments. We compare it against a “Structure-Only-EIG (Expected Information Gain)” ablation model, which is short-sighted and only seeks information about the causal structure at hand, analogous to previous models of active causal learning. Any learning about overhypotheses and future benefits would be incidental, rather than following deliberate choices to pick interventions with an eye toward overhypotheses and future learning. We anticipate our full model will produce better predictions than this ablation model. We also analyze the weight parameter of our model to investigate the degree to which people prioritize overhypothesis learning.

We test the ideas in our full hierarchical Bayesian model in two preregistered experiments. Our experiments are active learning extensions of Lucas and Griffiths ’s (2010) version of the “blicket detector” experiments (Gopnik & Sobel, 2000) and examine human behavior in a series of active causal learning tasks. Here participants have the opportunity to learn more efficiently in later tasks by learning and transferring causal overhypotheses from earlier tasks. We first checked the feasibility of our model’s ideas against qualitative patterns in participants’ interventions and judgments (Experiment 1). We then performed a more rigorous test of our model through a formal model comparison against ablation models and a random baseline (Experiment 2). We found our full hierarchical Bayesian model was the best at predicting interventions for the majority of individuals.

## Overall Experiment Design

**Fig. 3:**
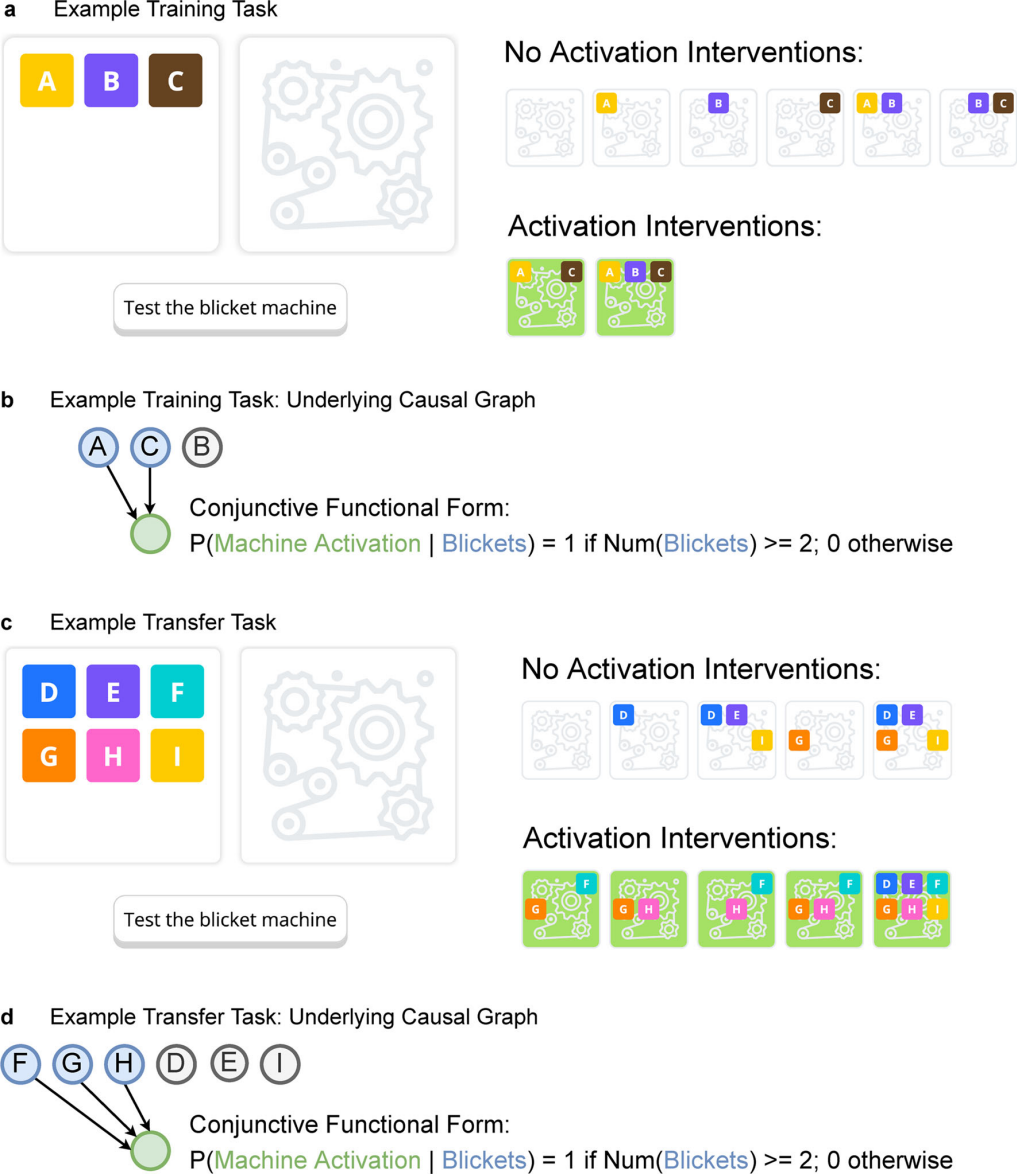
Example training and transfer tasks. **a**, Web interface for an example training task with 3 blocks (squares with colors and letters) and a blicket machine (embellished with cogs). An intervention involves clicking on blocks to set any combination on the machine and then pressing a button to test the machine’s response (activation with a green color or nothing). Interventions must always contain A and C to activate the machine (shown to the right). **b** Causal graph of the example training task: The causal structure defines A and C as blickets (causes). The functional form is conjunctive and defines the conditional probability of the machine’s activation (effect). **c** Web interface for an example transfer task with 6 blocks. Interventions must contain at least two of the blocks F, G and H to activate the machine (shown to the right; interventions are not comprehensive). **d** Causal graph of the example transfer task

Building on the general “blicket detector” paradigm (e.g., Gopnik & Sobel, 2000; Griffiths et al., 2011; Lucas et al., 2014; Sim & Xu, 2017; Kosoy et al., 2022) and especially Lucas and Griffiths ’s (2010) experimental setup, we presented participants with a task containing blocks (colored squares labeled with letters) and a “blicket machine”. We asked them to solve the causal learning problem of identifying “blickets” (causes) among the blocks (prospective causes) by observing the blicket machine’s binary response (effect). Whereas Lucas and Griffiths ’s study involved fixed sequences of events, ours used a computer-based web interface that allowed participants to actively produce their own sequences of events by choosing interventions (Fig. 3; see Appendix B for the instructions that we gave participants). An intervention involved putting any combination of blocks on the machine. The machine would then respond by activating or doing nothing. In order to choose informative interventions in this active blicket task, participants needed to consider their beliefs about both the causal structure (blicket identities) and the functional form (how the blicket machine activates in response to blickets). Participants encountered several active blicket tasks, where each one increased the level of difficulty and required increasingly selective interventions.

We chose our active blicket experiment design because it (1) was simple enough for online experimental participants to quickly understand, (2) was tractable to analyze with our hierarchical Bayesian model, (3) nonetheless required appropriate overhypotheses about the functional form to facilitate learning in future tasks, and (4) could be decomposed into a causal structure learning aspect and a functional form learning aspect. Within this design, we can formulate and test the ideas in our model: Participants’ interventions should not only yield information about the causal structure within each task, but also information about overhypotheses that can enable more efficient learning in future tasks.

Each active blicket task can be formalized as learning a causal graph (see Figs. 3 and 1). The *causal structure* of the graph defines what variables are causes and effects of other variables. The variables in the task are the blocks’ presence on the machine and the blicket machine’s activation. From the cover story, participants can easily identify the machine activation as the only plausible effect, but they do not know whether the other variables (blocks) are causes (blickets) or non-causes (non-blickets). Their goal is to solve the causal structure learning problem of identifying causes from non-causes, or blickets from non-blickets.

To identify blickets, participants must, however, also solve the *functional form* learning problem. The functional form defines the conditional probability of the effect (machine activation) given the causes that are present (blickets, not non-blickets, that are on the machine), and it is a function of these causes (blickets). For example, a disjunctive form says the conditional probability is 1 whenever at least one blicket is on the machine and 0 otherwise. A conjunctive form changes the blicket “threshold”, saying the conditional probability of a machine activation is 1 when at least two blickets are on the machine and 0 otherwise; see Table 1 for all the functional forms we consider. Under a disjunctive form, participants can intervene on one block at a time to identify whether that block is a blicket that activates the machine. However, under a conjunctive form, singleton interventions would not reveal anything about blickets. Participants would instead need to intervene on multiple blocks at a time to reveal any machine activations, and from there on, they might still need to narrow down which blocks in their intervention are actually blickets. Thus, in order to achieve the goal of identifying blickets, participants must learn both the causal structure and functional form of the task.

**Table 1:** Functional forms

Functional form	Interpretation		Sigmoid param.	
	Blicket threshold	Activation probability	Bias	Gain
Disjunctive	1	1	0.5	$\gg$ 1
Noisy Disjunctive	1	.75	0.9	11
Conjunctive	2	1	1.5	$\gg$ 1
Noisy Conjunctive	2	.75	1.9	11
3-Conjunctive	3	1	2.5	$\gg$ 1
Noisy 3-Conjunctive	3	.75	2.9	11

The functional form defines the conditional probability of the machine’s activation given the blickets (not non-blickets) that are on the machine, and it is a function of these blickets. In our experiment, the functional form can be interpreted as a rule that needs at least a threshold number of blickets to activate the blicket machine. At this threshold number, the activation occurs with some probability, but above the threshold, the activation always occurs. For example, with the noisy conjunctive form, the blicket machine activates with a.75 probability given a threshold of 2 blickets, but it always activates given 3 or more blickets. Each form has a corresponding sigmoid parameterization of bias and gain values

We presented participants with several active blicket tasks to investigate whether and how they would learn and transfer overhypotheses across these tasks. The earlier training tasks were designed so that participants could easily learn overhypotheses. For example, the first training task had only 3 blocks, allowing participants to intervene on all $2^3=8$ possible combinations within the constraints of the task (the 45 s time limit in Experiment 1, or the 12 intervention limit in Experiment 2). The final transfer task was then designed to measure the transfer of overhypotheses learned from training. The transfer task had more blocks (6 or 9) and thus was more combinatorially complex ($2^6 = 64$ or $2^9 = 512$ possible combinations), and it was no longer possible to intervene on all combinations of blocks within the task constraints. This complexity increased the importance of relying on previously learned overhypotheses to select just a few informative interventions.

Throughout our two experiments, we performed between-subjects manipulations of the blicket machine’s functional form in the training and transfer tasks, where all six functional forms we considered are listed in Table 1. We also manipulated the training length (one or two tasks). Our dependent measures were participants’ interventions and causal judgments in the transfer task. These measures would not only indicate whether participants’ interventions and judgments were informative of the transfer task’s causal structure and functional form, but also whether these were informative under overhypotheses about the functional form transferred from past training tasks.

## A Model of Actively Learning to Learn

Our hierarchical Bayesian model (preregistered at https://osf.io/vk9yd) represents causal beliefs at multiple levels of abstraction, including both lower-level beliefs about the causal structure and higher-level overhypotheses about the functional form. It infers the most likely causal structures and functional forms given the effects of different ensembles of blocks being placed on a blicket machine in the active blicket task. Each event is a pair (*q*, *o*) of the intervention *q* and the outcome *o*: the intervention is the set of blocks placed on the blicket machine, and the outcome is the binary response of the blicket machine (1 for activation or 0 for no activation). Given an event (*q*, *o*), the full joint Bayesian update for a particular structure $s \in S$ and particular form $f \in F$ is:

1 $$\begin{aligned} P(s,f|q,o) \propto P(q,o|s,f)P(s,f) \end{aligned}$$

Each causal structure *s* is represented by enumerating the set of blickets under this structure (e.g., {A, B} represents the causal structure where blocks A and B are blickets and any other blocks are non-blickets). The space of all causal structures *S* in an active blicket task is the power set of all blocks in that task, and we used a uniform prior over *S* for the start of each task. The space of functional forms *F* is described in the next section.

### Hypothesis 1: People Represent Rich Overhypotheses

Our model’s space of functional forms follows from our experiment’s cover story, which considers blickets as a general class of exchangeable objects that can have a generative effect (i.e., blicket machine activation). This means the functional form of the blicket machine’s activation should only consider individual blickets important to the extent that they contribute to the overall *number* of blickets that are on the machine: the functional form reduces to a function of the *number* of blickets. Furthermore, because blickets are generative causes, the form should output a conditional probability value that monotonically increases with the number of blickets. These properties can be satisfied by any monotonically increasing family of functions that maps the domain of zero and positive integers to the range [0, 1].

Therefore, following Lucas and Griffiths (2010), our model considers the *sigmoid* family of functional forms, evaluated at zero and positive integer inputs. This family is not only consistent with the exchangeability and generative properties of blickets, but it is also simple and able to express a rich space of forms with only two parameters, bias and gain. Variations of the bias and gain parameters roughly correspond to variations in blicket thresholds needed to produce an effect and noise levels of the effect, respectively. Expanding upon Lucas and Griffiths ’s (2010) space, we increase the range of bias and gain values to enable expressing 3-conjunctive forms. Our space now includes all the forms used in our experiment (see Table 1 for the bias and gain values of our experiment’s forms) as well as gradations between these forms (through finer variations of bias and gain values).

Formally, a particular form in the sigmoid family is fully described by a pair of bias *b* and gain *g* values. It outputs the conditional probability of the machine’s activation given the number of blickets *n* on the machine:

2 $$\begin{aligned} \text {sigmoid}(n) = \frac{1}{1 + e^{-(g(n - b))}} \end{aligned}$$

Our model’s space of functional forms covers combinations of $b \in [0, 2.85]$ and $g \in [0, 38]$ using a discrete grid of size 400 (the Cartesian product of 20 biases and 20 gains):

3 $$\begin{aligned} b\in & {} \{0.15i | i \in \mathbb {Z} \wedge 0 \le i \le 19\} \end{aligned}$$

4 $$\begin{aligned} g\in & {} \{2j | j \in \mathbb {Z} \wedge 0 \le j \le 19\} \end{aligned}$$

We chose joint priors over biases and gains that favor the kinds of functional forms people typically consider: disjunctive and reliable/nearly-deterministic forms (Lucas & Griffiths, 2010; Lu et al., 2008; Mayrhofer & Waldmann, 2016; Schulz & Sommerville, 2006). Rather than choosing a single prior, we chose a set of 24 priors that have these properties.

Our priors over biases and gains are gamma distributions parameterized by the shape and scale parameters. Rather than choosing a shape and scale directly, we found it more meaningful to choose a mode (${\text {scale}(\text {shape}-1)}$ for $\text {shape} > 1$) and scale, which respectively describe the most likely bias/gain value and the variability around that value.

For our bias priors, we considered 3 modes $\{0.3, 0.5, 0.8\}$ and 2 scales $\{0.1, 0.25\}$, creating 6 gamma distributions. For our gain priors, we considered 2 modes $\{10, 20\}$ and 2 scales $\{0.1, 1\}$, creating 4 gamma distributions. In combination, we created 24 joint priors over biases and gains.

We initialized and ran our model for each joint prior to produce predictions of people’s interventions under each prior. We then marginalized over these priors in our model comparisons (see “Cross-validation and Prior Marginalization”).

To test the scientific hypothesis that people represent rich overhypotheses, we compare to a *Fixed-Form* ablation model that removes this hypothesis. The Fixed-Form model is the same as our hierarchical Bayesian model except that it replaces our grid of sigmoid forms with a single deterministic ($g \gg 1$) and disjunctive ($b=0.5$) form.

### Hypothesis 2: People Transfer and Adapt Their Overhypotheses

After our model’s joint distribution over forms and structures is conditioned on events in one task, the posterior marginal distribution of functional forms is extracted and reused as the prior over functional forms for a new task. Our model then multiplies this transferred prior with a uniform distribution over the causal structures in the new task, creating a joint distribution for learning in the new task. Thus, our model predicts that people transfer and adapt their overhypotheses about functional forms.

The posterior marginal probability for a form *f* is:

5 $$\begin{aligned} P(f|q,o) = \sum _{s \in S} P(s,f|q,o) \end{aligned}$$

which is calculated from the joint posterior *P*(*s*, *f*|*q*, *o*) that also includes causal structures *S*.

Transferring the marginal distribution of functional forms is possible because the likelihood of the joint inference (Eq. 1) is calculated hierarchically: The likelihood is proportional to the conditional probability (of the effect) defined by the functional form (higher-level overhypotheses), which depends on the causal structure (lower-level beliefs) as an input. We abuse the functional form notation *f* to show this dependence in the likelihood:

6 $$\begin{aligned} P(q,o|s,f) \!=\! P(o|q,s,f)P(q|s,f) \propto {\left\{ \begin{array}{ll} f(|q \cap s|) &{} \text {if } o\!=\!1 \\ (1\!-\!f(|q \cap s|)) &{} \text {if } o\!=\!0 \end{array}\right. } \end{aligned}$$

where *P*(*q*|*s*, *f*) is a constant and *P*(*o*|*q*, *s*, *f*) can be rewritten in terms of the functional form *f*. The form *f* is a sigmoid function that returns the probability of activating the blicket machine ($o = 1$). Its input is the number of blickets (set cardinality) in an intervention *q* (a set of blocks on the blicket machine) according to the causal structure *s* (a set of blocks that are blickets under this structure).

Because the functional form can take in any causal structure, the same functional form can be used to compute likelihoods across distinct tasks with different causal structures (i.e., different numbers and identities of non-blickets and blickets). Thus, the same space of functional forms, along with its marginal distribution, can be transferred for learning across tasks.

To test the scientific hypothesis that people transfer and adapt their overhypotheses about the functional form, we compare to a *No-Transfer* ablation model that removes this hypothesis. The No-Transfer model is the same as our hierarchical Bayesian model, but it discards the part that reuses the marginal posterior over functional forms from a previous task as the prior in a new task. Instead, it reinitializes the prior over functional forms so that it is the same at the start of every task. We considered a set of 24 priors, which are described in “Hypothesis 1: People Represent Rich Overhypotheses”.

### Hypothesis 3: People Sacrifice Short-term Learning for Information Gain About Overhypotheses

Like previous computational accounts of active causal learning (e.g., Steyvers et al., 2003; Bramley et al., 2015; Coenen et al., 2015), our model considers an intervention’s expected information gain with respect to causal structures. Unlike previous accounts, our model additionally considers expected information gain with respect to overhypotheses about the functional form. Our model prefers interventions that maximize expected information gain on both structures and forms, predicting that people choose interventions that are not only informative for learning the causal structure but also for learning overhypotheses about the functional form.

Formally, the expected information gain (EIG) of an intervention *q* for a random variable *X* (functional forms *F* or causal structures *S*) is:

7 $$\begin{aligned} {\begin{matrix} &{} \text {EIG}_X(q) = \mathbb {E}[\text {I}(X;q,o)] = \mathbb {E}[\text {H}(X) - \text {H}(X|q,o)] = \\ &{} \sum \limits _{o \in \{0,1\}} \left[ -\sum \limits _{x \in X} P(x)\text {log}P(x) + \sum \limits _{x \in X}P(x|q,o)\text {log}P(x|q,o) \right] P(o|q) \end{matrix}} \end{aligned}$$

where I denotes information gain and H denotes entropy. The outer expectation is calculated with the probability of each outcome *o* for this intervention *q*. With this formulation, we note that information gain I is equivalent to mutual information and preferring higher expected information gain is tantamount to preferring lower expected conditional entropy.

Rather than calculating expected information gain on the joint distribution over causal structures and functional forms, our model uses a linear combination of their respective marginal expected information gains, weighted by parameter $w \in [0,1]$. This combined expected information gain (cEIG) of an intervention *q* is:

8 $$\begin{aligned} \text {cEIG}(q) = w\text {EIG}_F(q) + (1-w)\text {EIG}_S(q) \end{aligned}$$

This equation is only equivalent to joint information gain when form (*F*) and structure (*S*) are independent conditional on interventions and outcomes, which, here, they are not. However, we use this decomposition to approximate joint information gain because it serves the important purpose of allowing us to capture the possibility that people’s interventions preferentially maximize one kind of information over another. When $w=0$, our model collapses to the special case of only trying to learn about structure in the immediate task, as in previous models of active causal learning, while $w=1$ implies that only the functional form matters, as we might expect if people are primarily interested in learning overhypotheses for future use.

To reduce our model’s sensitivity to EIG scale differences, we first perform a min-max normalization for form EIGs and structure EIGs (across possible interventions *q*) before combining them in Eq. 8. This normalization is computed separately for each type of EIG as $\text {normalize}(x) = (x - x_{\text {min}}) / (x_{\text {max}} - x_{\text {min}})$. It ensures $\text {EIG}_F(q)$ and $\text {EIG}_S(q)$ are each within the range [0, 1].

To test the scientific hypothesis that people sacrifice short-term learning for information gain about overhypotheses, we compare our full model to a *Structure-Only-EIG* ablation model that removes this scientific hypothesis. The Structure-Only-EIG model sets $w=0$ (Eq. 8) in our hierarchical Bayesian model, predicting that interventions do *not* sacrifice short-term learning about causal structures. Instead, this ablation model only maximizes expected information gain about causal structures while learning overhypotheses incidentally. We also fit weights *w* to participants and analyze these fitted weights.

Overall, we compare all three ablation models described above with our full hierarchical Bayesian model. We compare them by how well they predict participant interventions, which we compute as the predictive likelihood of participant interventions under each model; “Predictive Likelihood” describes how we compute these by applying a softmax on combined EIGs. Additionally, we compare to a random baseline that samples interventions uniformly. For example, in a task with 6 blocks, the random baseline model would sample an intervention from $2^6=64$ possible combinations of blocks, producing a predictive likelihood of $\frac{1}{64}$ for each possible intervention.

## Experiment 1

In our first preregistered experiment (https://osf.io/n9cx2), we gauged the feasibility of our model’s ideas by testing a weaker version of these ideas: People choose interventions to learn overhypotheses about the functional form, which then enable more efficient learning in future tasks if these overhypotheses are appropriate (and vice versa). Before committing to testing our model through an extensive model comparison, we performed simpler analyses of qualitative patterns in participants’ interventions and causal judgments in active blicket tasks. We hypothesized that in a new task (called the *transfer* task), people would choose more efficient interventions and make more accurate judgments after training with the same functional form in past tasks. Conversely, they would choose less efficient interventions and make less accurate judgments after training with a different form. We also predicted that these effects would be larger if the same or different form was reinforced with more training tasks.

### Methods

#### Data Filtering

To represent the data accurately while accounting for potential data quality issues, we report results for both the full data set ($N=212{}$) and a filtered subset ($N_f=181{}$), which includes most (85.38%) of the original participants while requiring more participant engagement. The filtered participants made at least 9 interventions in the transfer task, which was the minimum number required to execute a straightforward strategy in the easier disjunctive variant of that task: testing whether each of the 9 blocks was a blicket that can individually activate the machine.

#### Participants

**Fig. 4:**
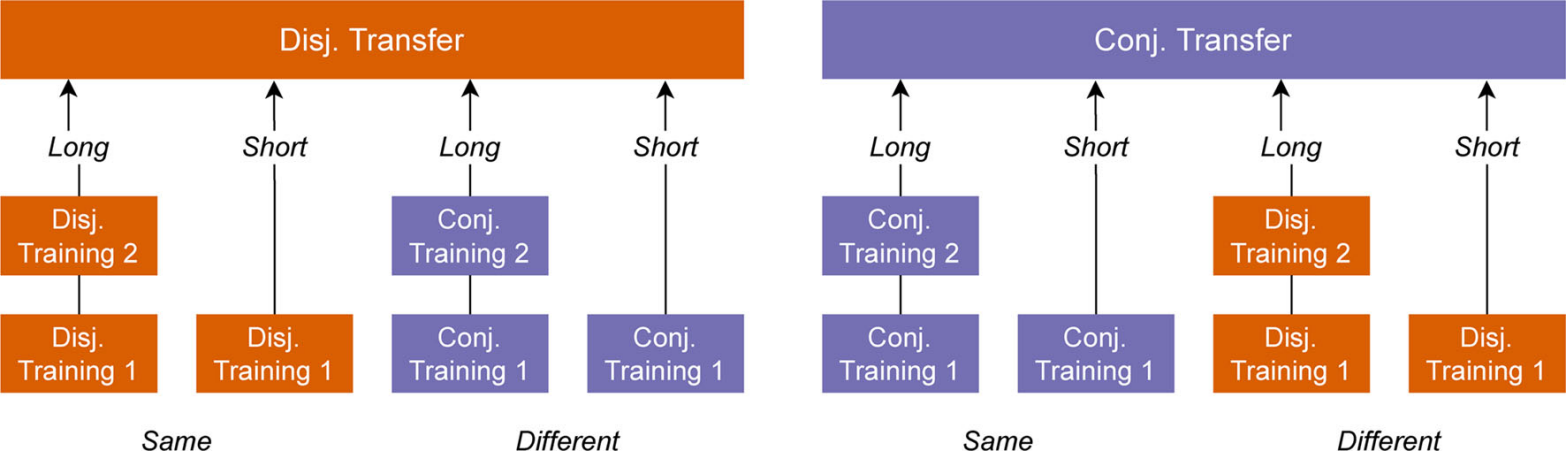
Experiment 1 conditions. Each of the 8 arrows represents a between-subjects condition and each box represents a training or transfer task. “Disj.” is short for Disjunctive and “Conj.” is short for Conjunctive. We manipulated the functional form of the transfer task (disjunctive or conjunctive), the training length (long with 2 training tasks, or short with 1 training task), and whether the training form was matched with the transfer form (same or different)

212 participants were recruited using Amazon Mechanical Turk (HIT Approval Rate $$\ge 99\%$$, Number of HITs Approved $$\ge 1000$$, Age $$\ge 18$$) for the 8 between-subjects conditions in Fig. 4. From left to right in this figure, the number of participants in each condition is 27, 29, 29, 26, 25, 25, 26, and 25. The corresponding numbers of *filtered* participants are: 23, 22, 22, 24, 23, 20, 25 and 22. Participants were paid $1.5 for completing the study (7.36 min on average, excluding the instructions) and received a bonus of up to $1.05 for their questionnaire performance, resulting in a mean total compensation of $2.32.

#### Procedure

Experiment 1 manipulated the functional form of the transfer task (disjunctive or conjunctive), whether this form was matched with their past training tasks (same or different), and training length (short or long, i.e., one or two training tasks before the final transfer task), creating 8 between-subjects conditions (Fig. 4).

Within each task, participants saw a web interface with blocks (colored squares labeled with alphabetical letters) and a blicket machine. Examples of this interface are shown and described in Fig. 3. Participants were asked to identify which of the blocks were blickets with the help of the blicket machine, and they were told the blocks’ colors, letters and positions did not reveal blickets. (Unknown to participants, we performed counterbalancing by randomizing the block color and whether a block was a blicket. Each block was labeled with a unique letter that was assigned in alphabetical order.) Participants could choose any number of interventions within a time limit of 45 s, where each intervention involved putting any combination of blocks on the machine. The machine would then respond by activating or doing nothing (according to a disjunctive or conjunctive functional form, which was unknown to participants). Participants could view their full history of interventions and machine responses.

Participants encountered a first training task with three blocks. If they were in a *long* training condition, they would encounter a second training task with six blocks followed by a final transfer task with nine blocks. Otherwise, if they were in a *short* training condition, they would directly move on to the transfer task without seeing the second training task. The number of blocks in each task is also listed in Table 2, along with how many of these blocks were blickets (unknown to participants). Even as the number of blocks (and along with it, the number of possible interventions) increased across tasks, the time limit remained at 45 s.

**Table 2:** Experiment 1: Number of blocks and blickets

Task	Num. Total Blocks	Num. Blicket Blocks
Training 1	3	1 (Disj.) or 2 (Conj.)
Training 2	6	3
Transfer	9	4

In each task, the number of blickets is contained within the total number of blocks. In the first training task, the “Disj.” (Disjunctive) variant has one blicket while the “Conj.” (Conjunctive) variant has two

Each training and transfer task was followed by a questionnaire with two types of binary causal judgments, one about identifying each block as a blicket or non-blicket (“Which blocks do you think are blickets?”), and another about predicting whether the blicket machine would activate in the presence of different combinations of blocks (“Will the blicket machine activate (light up with a green color)?”). Different combinations can have only blickets, only non-blickets, or a mix of both, where the ground truth is a mix of activation or no activation depending on the functional form and the number of blickets present (see Appendix B for more details).

Between tasks, participants only received feedback and associated bonus compensation for the correctness of their activation prediction judgments, not their blicket identification judgments. We used this feedback/compensation structure to limit what was revealed about the ground truth causal relationship, since only getting feedback about whether or not a combination of blocks activated the machine would not reveal much about which of those blocks were blickets. Instead, participants would need to rely on their own interventions to identify blickets. Furthermore, the compensation would incentivize participants to make more accurate judgments, which meant they also needed to make more informative interventions.

### Results

#### Causal Judgments

First, we performed a preregistered analysis (https://osf.io/n9cx2) of participants’ causal judgments in the transfer task, where they aimed to identify which of the 9 blocks were blickets. Participants also judged whether the blicket machine would activate in response to different combinations of blocks. These judgments are described in Appendix A, and were broadly consistent with blicket identification judgments. Here we focus on the latter because the blicket identification format follows more closely from past blicket studies (Lucas & Griffiths, 2010).

**Fig. 5:**
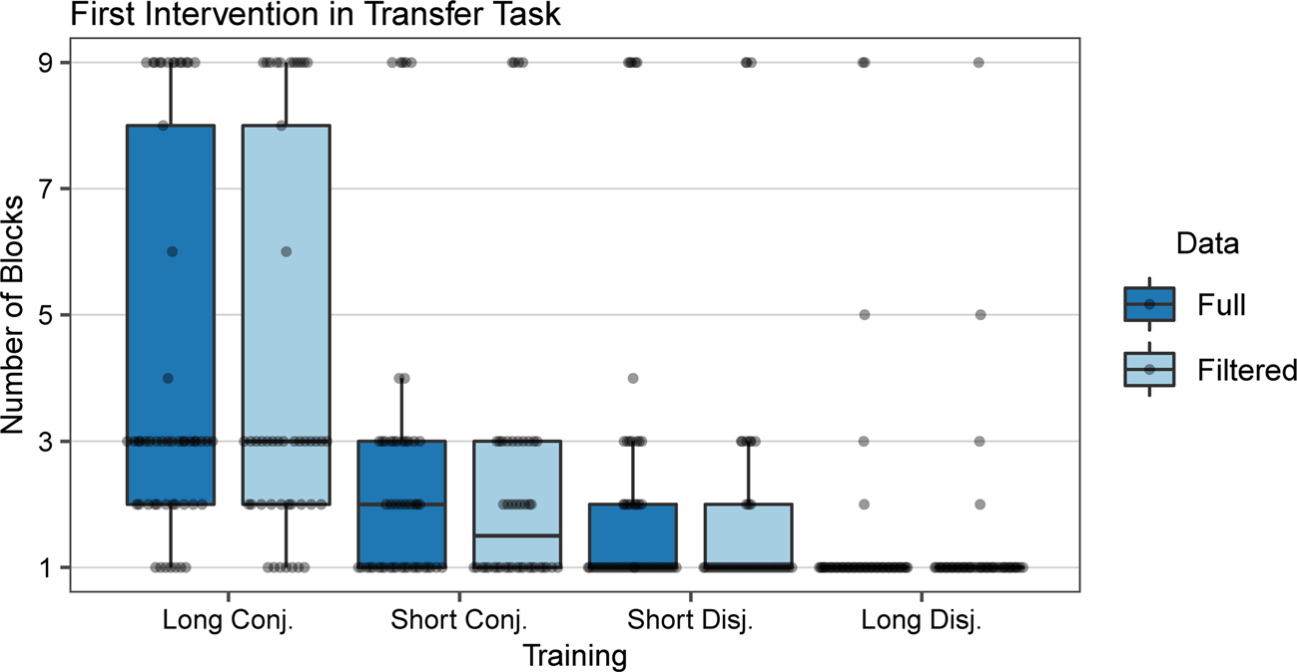
Experiment 1: Number of blocks in the first intervention in the transfer task. This is plotted against the functional form of past training tasks and the training length. The box-and-whisker plots show the quartiles of the full or filtered data; each overlaid point represents a participant

We expected causal judgment accuracies to improve in conditions where the transfer and training tasks had matching functional forms, as compared to conditions with mismatched forms. We used Welch t-tests (two-tailed) to investigate the effects of match between pairs of conditions. (For a visual comparison of mismatched and matched conditions, see Appendix A Fig. A1a.) In the disjunctive transfer conditions, the comparisons were mostly consistent with our expectations: in the full data, the mean blicket identification accuracy showed a trend toward improvement from mismatched (conjunctive training) to matched (disjunctive training) conditions with long, $t(47.82)=-2.00, p=.051$, and short training, $t(49.76)=-1.80, p=.078$. These trends were significant in the filtered data where participants were more engaged (long: $t_f(42.10)=-2.18, p_f=.035$; short: $t_f(43.99)=-2.47, p_f=.017$).

In the conjunctive transfer conditions, however, the difference between matched (conjunctive training) and mismatched (disjunctive training) accuracies was non-significant. We suspected this weaker effect was due to the conjunctive transfer task being too difficult to learn, regardless of training match and length. This suspicion was supported by the results in our next experiment, where we lowered the difficulty of the conjunctive transfer task and found a significant improvement from mismatched to matched conditions (see Appendix A). Finally, our Welch t-test results above are largely consistent with additional analyses of the match effect together with the effects of training length and the transfer task’s form; we report these analyses in Appendix A.

**Fig. 6:**
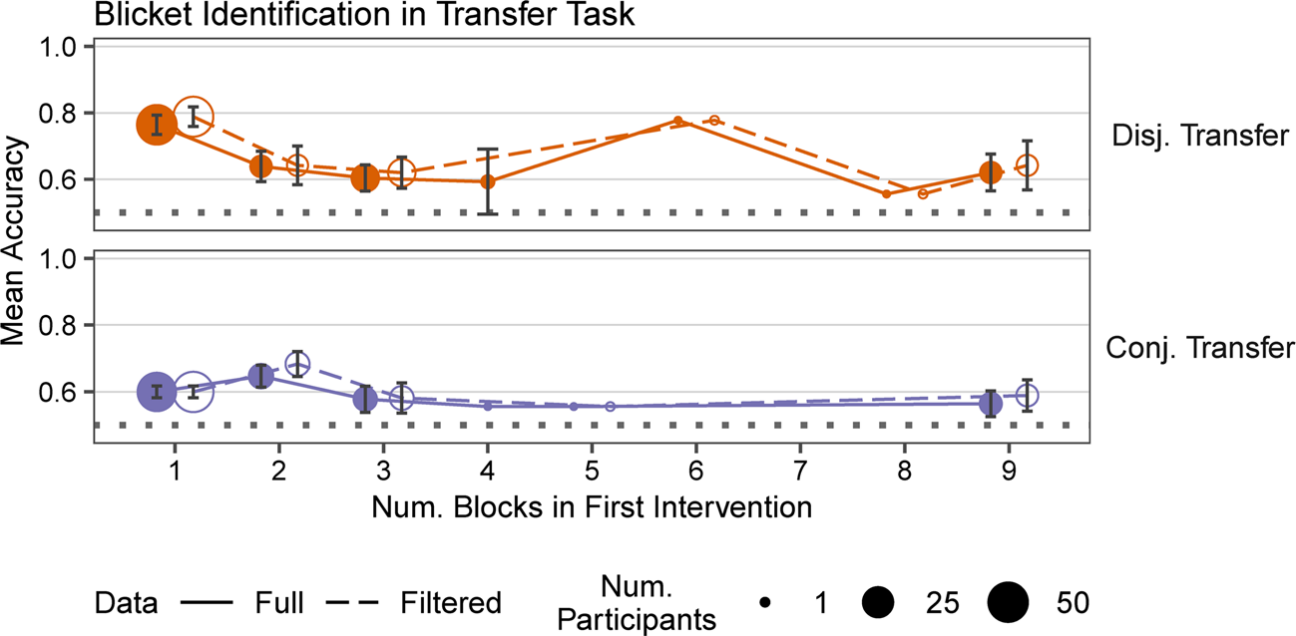
Experiment 1: Mean participant accuracies for blicket identification questions in the transfer task. This is grouped by the number of blocks in the first intervention and the transfer form. For a particular number of blocks, the size of the dot represents how many participants are involved in calculating the mean accuracy. The mean is calculated separately for the full (solid lines) and filtered (dashed lines) data. Chance (.5) accuracy is shown with a dotted gray line. Error bars in either direction denote the magnitude of the standard error but are omitted for points with a single participant, where the standard error is undefined

#### First Intervention

Then we analyzed participants’ *first* intervention in the transfer task. The first intervention had to be chosen before participants learned anything about the functional form in the transfer task, making it a simple marker of whether participants’ interventions were informative under a functional form from their past training. Under a disjunctive form, intervening on a single block would be informative for identifying blickets, requiring only nine interventions in all. In contrast, this singleton intervention would be completely uninformative under a conjunctive form, which would require intervening on more blocks at a time to identify blickets. We expected that participants’ first intervention in the transfer task would be informative under their training form, and therefore, we also expected this intervention to be more efficient if the training and transfer forms were the same.

We performed t-tests to gauge whether the first intervention in the transfer task was informative under the training form: The mean number of blocks in the first intervention was significantly higher after conjunctive training (short and long) than after disjunctive training (short and long), $t(183.39)=4.62, p<.001$ (filtered: $t_f(144.96)=4.75, p_f<.001$), suggesting participants’ interventions were informative under their training form. These differences are also visualized in Fig. 5, and are largely consistent with our further analyses in Appendix A, where we analyze the training form together with training length.

To understand when the first intervention would be efficient for learning in the transfer task, we fitted a (binomial) logistic regression model to predict blicket identification accuracy in the transfer task (9 trials). The predictors included the number of blocks in the first intervention, the functional form of the transfer task, and their interaction. There was a significant main effect of the transfer form ($z=4.26, p<.001$; filtered: $z_f=4.90, p_f<.001$), underscoring the relative difficulty of the conjunctive condition, and no significant main effect for the number of blocks (all $p \ge .382$), suggesting that any effect of the number of blocks was not due to choosing more (or fewer) blocks being a better general-purpose policy. Rather, the effect of the number of blocks was due to being informative of a particular transfer form: this interaction did not reach significance in the full data ($z=-1.73, p=.084$), but was significant for the more engaged participants in the filtered data ($z_f=-2.02, p_f=.043$). Specifically, Fig. 6 shows the disjunctive accuracy peaks at a singleton block and decreases as the number of blocks increases, while the conjunctive accuracy peaks at two blocks.

**Fig. 7:**
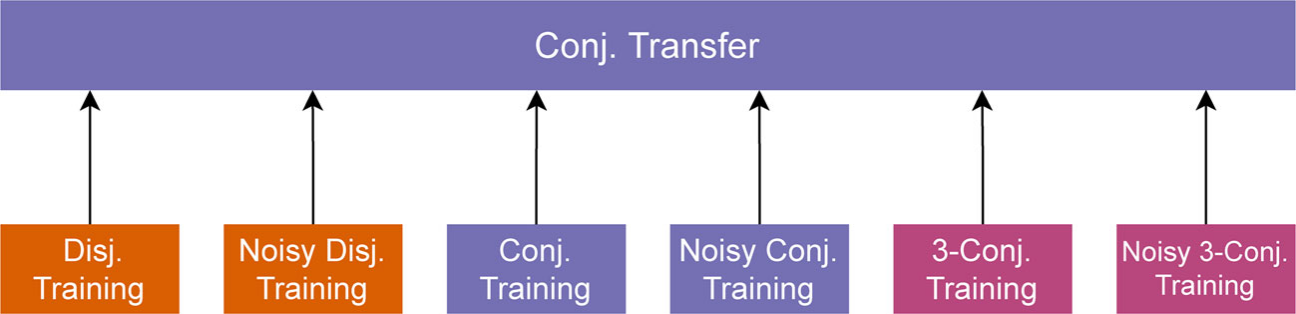
Experiment 2 conditions. Each of the 6 arrows represents a between-subjects condition and each box represents a training or transfer task. “Disj.” is short for Disjunctive, “Conj.” is short for Conjunctive, and “3-Conj.” is short for 3-Conjunctive. We fixed the transfer task’s form as conjunctive and manipulated the functional form of the training task. For the training form, we varied both the minimum number of blickets needed to activate the blicket machine—1 (disjunctive), 2 (conjunctive), or 3 (3-conjunctive)—and whether that activation was deterministic or noisy (probability 0.75 of activation given the minimum number of blickets)

Putting together the results on the first transfer intervention, participants chose interventions that tended to be informative under their training form: they chose fewer blocks after disjunctive training than after conjunctive training, with a focus on singleton blocks after disjunctive training (Fig. 5’s median lines). This first intervention was consistent with more efficient learning in a matched transfer task: a singleton block indicated better blicket identification in the disjunctive transfer task and two blocks indicated better blicket identification in the conjunctive transfer task. Combined with the results on participants’ causal judgments, Experiment 1 supports our hypothesis that, in the new transfer task, people choose more efficient interventions and make more accurate causal judgments after training with the same functional form. These patterns suggest people learn and transfer overhypotheses about the functional form, and they are able to exploit these overhypotheses to improve their active learning in similar future situations. Thus, Experiment 1 provides initial support for the ideas in our hierarchical Bayesian model, focusing on a weaker version of these ideas in exchange for being amenable to simpler statistical analyses. These results motivate us to commit to an expensive model comparison in Experiment 2, which enables us to test our model’s ideas more rigorously.

## Experiment 2

While Experiment 1’s results support the view that people adapt their interventions in ways that are consistent with having learned overhypotheses, we did not find significant improvements in conjunctive transfer task performance from mismatched to matched conditions. Under the logic that this lack of improvement may have been due to the conjunctive transfer task being too difficult, and to make a systematic model comparison feasible, we preregistered and conducted a second experiment (https://osf.io/vk9yd; $N = 250{}$). Experiment 2 lowered the conjunctive transfer task difficulty by reducing the number of blocks from 9 to 6. This lower number of blocks also enabled a computationally tractable evaluation of our main and ablation models.^2^

Experiment 2 also included a wider range of conditions that improved our ability to evaluate our models and test our scientific hypotheses (Fig. 2). Compared with Experiment 1, Experiment 2 expanded the manipulation of the training functional form to be representative of the rich space considered by our model and to test whether participants were sensitive to this space (Hypothesis 1). We varied both the minimum number of blickets needed to activate the blicket machine—1 (disjunctive), 2 (conjunctive), or 3 (3-conjunctive)—and whether that activation was deterministic or noisy (probability 0.75 of activation given the minimum number of blickets), creating 6 between-subjects conditions (the training length was fixed to a single task; see Fig. 7). The transfer task was fixed to have the same deterministic conjunctive form for all 6 between-subjects conditions, which tested how participants’ interventions in the same task would differ depending on overhypotheses learned from past training tasks. No matter if the training form was a mismatched (noisy) disjunctive form, a matched (noisy) conjunctive form, or a similar (noisy) 3-conjunctive form, we wanted our model to capture how participants transferred overhypotheses about the training forms (Hypothesis 2). Finally, the transfer task’s complexity and intervention limit required more efficient learning of both the causal structure and functional form, testing how participants would pick more informative interventions and whether these interventions would not only focus on the structure but also on overhypotheses about the form (Hypothesis 3).

We evaluated the hypotheses formalized by our model in a model comparison against ablation models, where models were ranked by how well they predicted participant interventions. The ablation models each removed one hypothesis at a time, so comparing against them allows us to test how important each hypothesis is for predicting participant interventions.

### Methods

#### Participants

250 participants were recruited using Amazon Mechanical Turk (HIT Approval Rate $$\ge 99\%$$, Number of HITs Approved $$\ge 1000$$, Age $$\ge 18$$) for the 6 between-subjects conditions in Fig. 7. From left to right in this figure, the number of participants in each condition is 42, 40, 46, 41, 40, and 41. Participants were paid $1.28 for completing the study (12.38 min on average, excluding the instructions) and received a bonus of up to $1.22 for their questionnaire performance, resulting in a mean total compensation of $1.99.

#### Procedure

Experiment 2 manipulated the functional form of the training task using all 6 forms in Table 1, creating 6 between-subjects conditions (Fig. 7). The transfer task’s functional form was fixed to the conjunctive form.

The rest of the procedure was the same as Experiment 1 except for some adjustments to the training length, transfer task complexity, task constraints, and questionnaires. These differences are described below.

While Experiment 1 used either one or two training tasks, Experiment 2 fixed the training length to a single training task. The transfer task’s complexity was also reduced from 9 blocks to 6. This reduction addressed how a conjunctive transfer task with 9 blocks was likely too difficult to reveal whether participants’ judgments were improving with matched overhypotheses (see Appendix A). See Table 3 for the number of blocks in each task, as well as how many of those are blickets.

**Table 3:** Experiment 2: Number of blocks and blickets

Task	Num. total blocks	Num. blickets	Functional form
Training	3	1	Disjunctive
		1	Noisy Disjunctive
		2	Conjunctive
		2	Noisy Conjunctive
		3	3-Conjunctive
		3	Noisy 3-Conjunctive
Transfer	6	3	Conjunctive

In each task, the number of blickets is contained within the total number of blocks, and when these two numbers are the same, then all blocks are blickets. The training task always has three total blocks, but the number of blickets varies with the functional form

The constraint in each task was changed from a time limit (with a variable number of interventions that depended on the participant) to a fixed number of interventions: 12 in the training tasks, and 20 in the transfer task. Both numbers of interventions have been verified as sufficient under our hierarchical Bayesian model (see preregistration: https://osf.io/vk9yd).

Following each task, the questionnaire had the same content as Experiment 1—blickets and the blicket machine—but differed in its exact format: Participants were asked to rate blocks as blickets on a 0–10 scale (rather than make a binary blicket/non-blicket judgment like in Experiment 1) and to create 5 examples for teaching others about the blicket machine. The details are explained in our preregistration (https://osf.io/vk9yd); we do not cover these details here because our analysis of Experiment 2 focuses on model evaluation rather than the questionnaire. Participants received bonus compensation for the correctness of their blicket ratings and the informativeness of their teaching examples, but did not receive any feedback (including their accumulated amount of bonus compensation) about their answers until after the experiment. As in Experiment 1, this feedback structure required participants to learn from only their own interventions and incentivized them to choose more informative interventions.

### Model Comparison

We performed a preregistered comparison (https://osf.io/vk9yd) of our hierarchical Bayesian model, the ablation models and the random baseline model, ranking them by how well they predicted participant interventions in Experiment 2’s transfer task.

#### Predictive Likelihood

To evaluate how well each model predicted participant interventions, we calculated the predictive likelihoods of participant interventions under that model. This predictive likelihood is the probability that the model would have chosen that intervention. Thus, if a model assigns higher predictive likelihoods to participant interventions, it is better at predicting the participant’s intervention choices.

Our hierarchical Bayesian model and the ablation models assign a predictive likelihood to participant *p*’s intervention $q_p$ through the following steps: (1) The model’s joint distribution over forms and structures is conditioned on participant *p*’s history of interventions and outcomes (up to and not including $q_p$) in the current task. Note that, except for the No-Transfer ablation model, a model’s joint distribution carries over the posterior over functional forms learned from *p*’s interventions and outcomes in previous tasks (see “Hypothesis 2: People Transfer and Adapt Their Overhypotheses”). (2) The history-conditioned joint distribution is then used to compute $q_p$’s combined expected information gain (for both causal structures and functional forms; see Eq. 8), which we abbreviate as cEIG. (3) Finally, following work showing that people *stochastically* prefer choices close to optimal (McFadden, 1973), we use a softmax function $\sigma$ to assign a probability/likelihood for selecting $q_p$ based on its cEIG. The predictive likelihood of $q_p$ is thus:

9 $$\begin{aligned} \sigma (\text {cEIG}(q_p)) = \frac{e^{\frac{1}{t}\text {cEIG}(q_p)}}{\sum _{i} e^{\frac{1}{t}\text {cEIG}(q_i)}} \end{aligned}$$

where *t* is the temperature parameter of the softmax function. *i* enumerates all the *possible* choices for that intervention, as opposed to (but does include) the actual choice $q_p$ made by the participant. In Experiment 2’s transfer task, a participant had 64 *possible* choices per intervention, corresponding to all the possible combinations of blocks.

The softmax temperature *t* controls how sensitive the model is to predicting (i.e., assigning higher predictive likelihoods to) intervention choices that have higher cEIG: lower temperatures increase the model’s preference for intervention choices that maximize cEIG, while higher temperatures decrease the model’s preference for any intervention choice, making the model tend toward assigning a uniform predictive likelihood to all possible intervention choices.

The random baseline model samples interventions uniformly, so it assigns a fixed predictive likelihood of $\frac{1}{64}$ to every participant intervention in Experiment 2’s transfer task. 64 is the number of possible choices for any intervention, corresponding to all the possible combinations of blocks.

#### Cross-validation and Prior Marginalization

In order to calculate the predictive likelihood under our model and the ablation models (and not the random baseline model), two parameters need to be fitted: the softmax temperature *t* and the weight *w* in the combined expected information gain (see Eq. 8). It is not clear what kinds of temperatures and weights would be appropriate for predicting people’s behavior, so we fit them to participant interventions using the training folds in cross-validation, which is a standard method for model fitting and evaluation (Gelman et al., 2013). We considered all temperature-weight combinations from the values below:

$$\begin{aligned} t&\in \{0.001, 0.01, 0.1, 1, 10, 100\} \\ w&\in \{0.1, 0.2, 0.3, 0.4, 0.5, 0.6, 0.7, 0.8, 0.9, 1.0\} \end{aligned}$$

The parameterization $w=0$ (not listed above) means that a model only maximizes expected information gain about causal structures while learning about functional forms incidentally. This parameterization is the *only* weight considered for two of our ablation models: The *Fixed-Form* model assumes a single functional form, meaning that learning and information gain only happen in the space of causal structures. The *Structure-Only-EIG*, by definition, only cares about causal structure learning and goes against the scientific hypothesis that people sacrifice such learning for information gain about overhypotheses. Since our remaining models do not have such commitments, they do not consider the $w=0$ parameterization but instead have a fitted weight from the possible values above.

For each model, we performed two kinds of cross-validation evaluations: an *averaged* evaluation over all participants ($N=250$) and interventions (20 per participant) in Experiment 2’s transfer task, and an *individual differences* evaluation that only considered one participant’s interventions at a time.

In the averaged evaluation, participants were randomly split into four balanced folds. Holding out one fold at a time, we fitted a model’s parameters by maximizing the mean predictive likelihood in the remaining folds, where this mean is first calculated for each functional form prior (see “Hypothesis 1: People Represent Rich Overhypotheses”) and then marginalized over all 24 priors using a uniform distribution. (This marginalization is not applicable to the first ablation model, which only has a single prior with a single disjunctive form.) We then used these fitted parameters to evaluate the model’s mean predictive likelihood in the hold-out fold with the same process for marginalizing over priors. The mean of all four hold-out evaluations was used for comparing models, where higher values meant a model was a better predictor of average participant intervention strategies.

In the individual differences evaluation, the cross-validation process was the same as the averaged one except it was performed within each individual participant. For each participant, their 20 interventions were split into four balanced folds and the mean predictive likelihood of all hold-out evaluations was used for selecting the best model for that participant. If a model was the best predictor for a higher number of participants, then that model was a better predictor of individual intervention strategies.

### Results

We conducted two analyses to test our model’s three hypotheses. The first analysis seeks to understand which single model explains the totality of participants’ decisions, assuming all participants approach the task in the same way. Our second analysis seeks to understand how many participants are behaving in accordance with each model, under the assumption that individuals might vary, e.g., in how much they prioritize information about the functional form and their propensity to transfer information from one task to the next.

In Experiment 2’s transfer task, we compared our model’s predictive likelihoods to the random baseline and the ablation models. To perform well, our model needed to make specific predictions about which combinations of blocks were good interventions among $2^6=64$ possible combinations of 6 blocks, and these predictions needed to align with each of the 20 interventions chosen by each participant.

#### Best Single Model

Our averaged evaluation takes the mean over predictive likelihoods computed for every participant intervention (in hold-out folds of the cross-validation process explained above), comparing models by how well they predicted the entire set of participants. The No-Transfer ablation model had the highest mean predictive likelihood ($\text {mean (M)} \pm \text {standard error (s.e.)} =.059 \pm .002$) and was closely followed by our full model ($\text {M} \pm \text {s.e.} =.055 \pm .003$). All other models had lower predictive likelihoods (Fixed-Form: $\text {M} \pm \text {s.e.} =.018 \pm .000$; Structure-Only-EIG: $\text {M} \pm \text {s.e.} =.018 \pm .002$; Random (fixed value: .016)). It is likely that the No-Transfer model performed better than our full model because it has a more diffuse prior, a point that we expand on in the Discussion (“Individual Differences vs. Average”).

#### Individual Differences

**Fig. 8:**
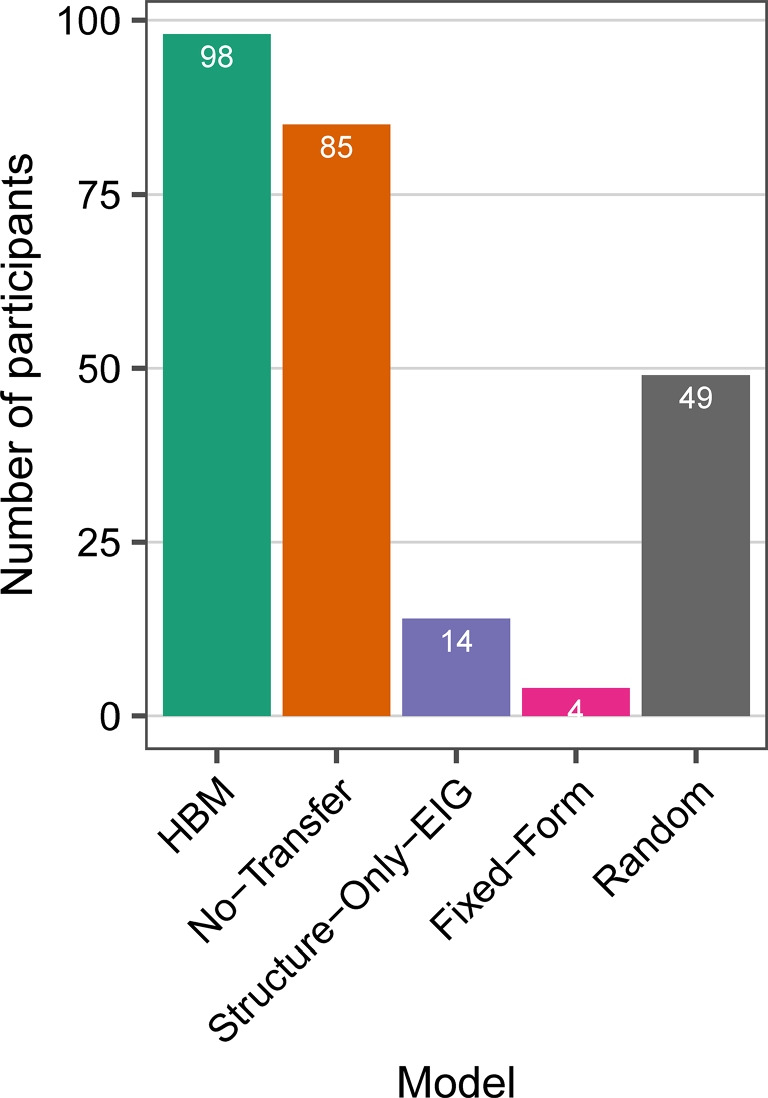
Best model for individual participants (Experiment 2). Each model’s bar counts the number of participants whose transfer task interventions were best-predicted by that model, i.e., assigned the highest mean predictive likelihood by that model compared with all other models. The comparison was performed on a per-participant basis and using cross-validation and marginalization over priors. Our Hierarchical Bayesian Model (HBM) was the best predictor for the highest number of participants compared with the ablation models (No-Transfer, Structure-Only-EIG (Expected Information Gain), Fixed-Form) and a random baseline

In the individual differences analysis, we compared all models on a *per-participant* basis (using cross-validation within individuals, as explained above) and distributed participants by their individually best model. Here we found that our full model was the best predictor for the highest number of participants (Fig. 8). The No-Transfer model captured the second-highest number of participants, which may be due to these participants having a strong prior for disjunctive and deterministic relationships and treating contradictory training evidence (e.g., conjunctive or 3-conjunctive evidence) as rare occurrences. We elaborate on this point in the Discussion (“Individual Differences vs. Average”).

**Fig. 9:**
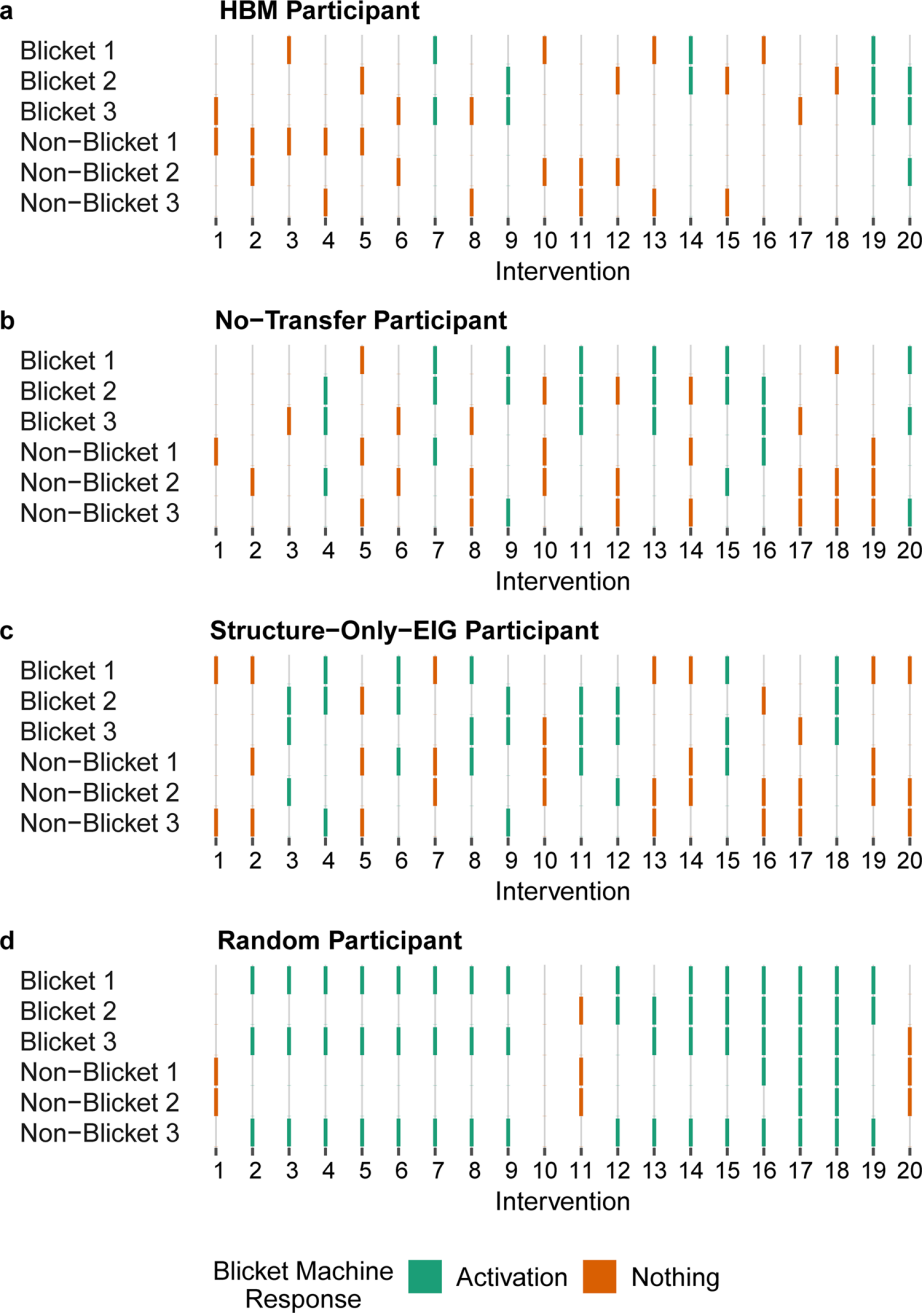
Representative individual participants for each model (Experiment 2). Their 20 interventions are shown for the conjunctive transfer task and they had previously completed a matched conjunctive training task (i.e., they all belonged to the conjunctive training condition). Each intervention contains some combination of the transfer task’s 6 blocks, whose identities (blicket or non-blicket) are labeled in the figure but were not known to participants. The blicket machine’s response (activation or nothing) is marked with the color and was also known to participants. The Fixed-Form ablation model is not included because it was not the best predictor for any participant in the conjunctive training condition

To provide intuition for the different models and what kinds of individuals they capture, we plot a representative individual for each model in Fig. 9. This figure shows participants from only the conjunctive training condition and their 20 interventions in the conjunctive transfer task (see Fig. 3 for examples of these conjunctive training and transfer tasks). Looking at the same training condition makes it possible to tease apart individual differences that are captured by differences in the models rather than differences in participants’ past training experience.

Our hierarchical Bayesian model (HBM) best-predicted a participant who immediately began the transfer task by testing pairs of blocks (Fig. 9a), suggesting they transferred conjunctive-favoring overhypotheses and initially focused on causal structure learning. It appears that they were searching for pairs of blickets that could activate a conjunctive blicket machine, starting by testing all pairs involving the “Non-Blicket 1” block (interventions 1–5; the non-blicket label is unknown to the participant) to identify whether this block was a blicket. Their interventions also appeared to be informative about the functional form: They had a good coverage of various pairs of blocks, and, if any pair resulted in an activation, they would be able to identify conjunctive forms as being more likely than 3-conjunctive ones. And after finding that “Blickets 1, 2, 3” activated the machine together in pairs (interventions 7, 9, 14), they further checked that disjunctive forms were unlikely by testing these blickets in isolation (interventions 16–18). Thus, all of our model’s assumptions were crucial for predicting this participant’s intervention strategy.

The ablation and random models captured other qualitatively distinct strategies. The No-Transfer ablation model best-predicted a participant who began with singleton interventions before intervening on multiple blocks (Fig. 9b). This strategy suggests they adapted their beliefs and interventions to consider forms beyond disjunctive ones, but they were initially trying to identify the causal structure under disjunctive forms. This behavior is consistent with the disjunctive-favoring priors that people tend to bring to the blicket experiment (Lucas & Griffiths, 2010), rather than overhypotheses transferred from the previous conjunctive training task (i.e., ablating our full model’s assumption that people transfer their overhypotheses).

**Fig. 10:**
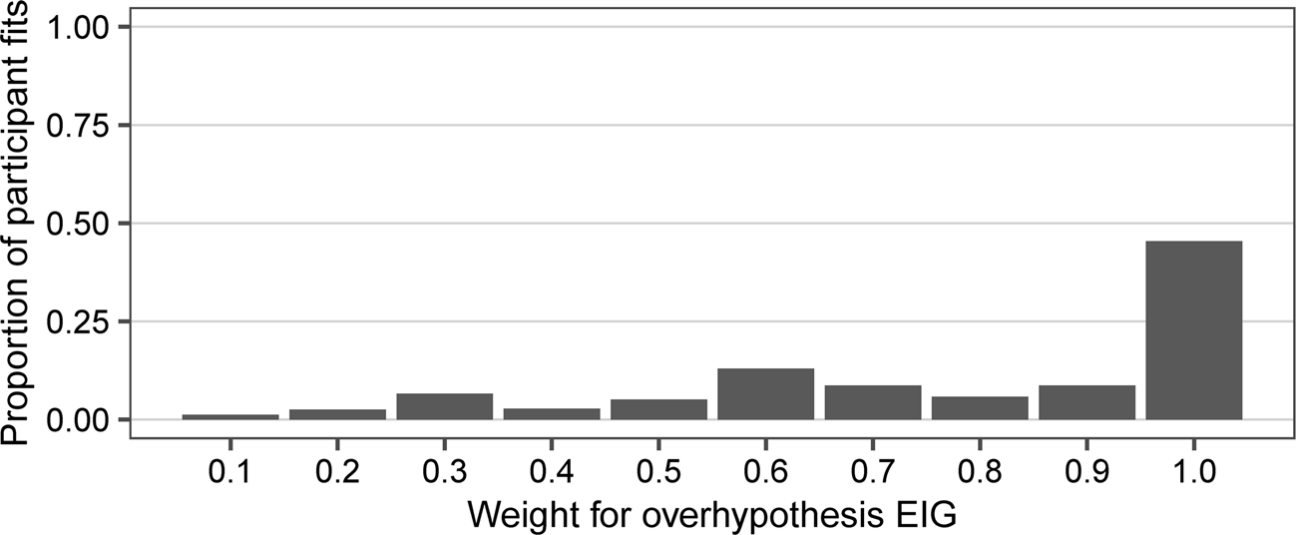
Weighting of overhypothesis vs. causal structure EIG (Experiment 2; HBM participants). The histogram is a distribution of weights fitted to individual participants’ intervention choices and observations. Higher weights in our model mean having more focus on maximizing overhypothesis EIG and less on maximizing causal structure EIG (Eq. 8). 81.63% of fits have $\text {weight}>0.5$ (i.e., $\text {structure weight}<0.5$), suggesting participants’ intervention choices sacrifice a substantial amount of information gain about causal structures in favor of information gain about overhypotheses

The Structure-Only-EIG ablation model best-predicted a participant whose interventions were consistent with having learned conjunctive-favoring overhypotheses from training, transferring these overhypotheses to the transfer task, and then prioritizing learning causal structure under these overhypotheses: They focused heavily on multiple-block interventions, suggesting they were searching for blickets under conjunctive-favoring overhypotheses (Fig. 9c). However, all but one of their interventions tested triplets of blocks and none tested singletons. This strategy seems inefficient for identifying whether all three blocks were needed for an activation (3-conjunctive), or only a subset of two (conjunctive) or one (disjunctive) was needed. Thus, they appeared to care little about disambiguating forms (i.e., ablating our full model’s assumption that people seek information about overhypotheses).

The Fixed-Form ablation model was not the best predictor for any participant in the conjunctive training condition. This result suggests that, in the conjunctive training and transfer tasks, participants considered alternative forms beyond a single disjunctive functional form (i.e., keeping our full models’ assumption of representing a rich overhypothesis space).

Finally, the random baseline model best-predicted a participant whose interventions were not compatible with other models (Fig. 9d). This participant appeared to employ a low-effort strategy where they were trying to complete the transfer task quickly (perhaps to receive their performance-independent compensation) rather than learn the task, a point which we cover in the Discussion. A possible low-effort strategy is the positive testing strategy (see the Introduction for more discussion of this strategy). As opposed to our model’s commitment to discriminating between many possibilities, this strategy is marked by choosing interventions that favor positive evidence (i.e., activating the blicket machine) for confirming a single hypothesis. It is possible some Random model participants were employing this strategy: They have a mean of 12.76 positive outcomes ($$\pm .80$$ standard error) out of 20 tests (63.80%), which is more than a purely random policy with 10 expected positives (50%). Their mean number of positives is also more than all other participants’ mean of 6.35 positives ($$\pm .24$$ standard error; 31.75%), with this difference being statistically significant, $$t(56.89)=7.64,$$$$p <.001$$.

##### Weighting Overhypotheses vs. Causal Structures

We now take a closer look at the individuals best-predicted by HBM. HBM commits to treating overhypothesis learning as being useful and transferable in the long term, and to gaining information about such overhypotheses at the expense of short-term learning about causal structures. This preference for overhypothesis learning is represented as a positive weighting for overhypothesis EIG in HBM’s (soft-) maximization of a linear combination of overhypothesis EIG and causal structure EIG (Eqs. 8 and 9). To better understand the extent of this preference, we distributed the positive weight values fitted to HBM participants in Fig. 10.^3^

Here we found that 81.63% of fits have overhypothesis weight $>0.5$ (i.e., $\text {structure weight}<0.5$), which corresponds to the overhypothesis EIG term dominating over the causal structure EIG term in HBM’s maximization objective. Interestingly, 45.41% of fits have an overhypothesis weight of 1, implying a causal structure weight of 0. However, it is unlikely that participants do not care about learning causal structures at all. A weight of 1 may instead result from participants seeking information about structures when that does not entail sacrificing much information about overhypotheses. And when there is a clear disagreement between structure EIG and overhypothesis EIG, participants may favor the latter. In summary, Fig. 10 suggests participants sacrifice a substantial amount of causal structure learning in favor of seeking information about overhypotheses.

Overall, participants employed several qualitatively different strategies in the transfer task, even when their previous training was the same (Fig. 9). To accommodate this variability, we performed an individual differences analysis to find the best model per participant. The largest group of individuals employed a strategy that was best-predicted by our hierarchical Bayesian model, suggesting our model captured people’s dominant intervention strategy. This group of individuals also have fitted weights that suggest they sacrifice a considerable amount of causal structure learning in favor of overhypothesis learning (Fig. 10).

## Discussion

How do people actively learn to learn causal relationships? That is, how and when do people choose interventions that facilitate long-term learning and promise more informative future interventions? We proposed and tested a hierarchical Bayesian model that goes beyond past models of active causal learning by representing people’s beliefs at multiple levels of abstraction. The lower-level beliefs are about the causal relationship in front of them, while the higher-level beliefs, or overhypotheses (Goodman, 1955; Kemp et al., 2007), are about general causal properties that generalize to future situations and shape future inferences. We focused on overhypotheses about the functional form, which governs how multiple causes combine or interact to produce an effect. Our model formalizes three key hypotheses: (1) people represent rich overhypotheses; (2) people transfer and adapt their overhypotheses across tasks; and (3) they sacrifice short-term learning for information gain about overhypotheses.

We tested how well our model explained human behavior in our two active blicket experiments, where we used a total of 14 between-subjects manipulations of training and transfer tasks to probe how and when people choose interventions that facilitate long-term learning across those tasks. We found initial support for our model in qualitative patterns of participant behavior (Experiment 1), where their interventions and judgments showed long-term improvement when the training and transfer tasks had the same functional form. These results motivated us to perform a more extensive test of our model using an individual differences-based model comparison (Experiment 2), which demonstrated our model was the best predictor for the largest group of participants. Our results suggest that when there are abstract similarities across active causal learning problems, people readily learn and transfer overhypotheses reflecting these similarities. Moreover, people exploit these overhypotheses to facilitate long-term active learning.

### Individual Differences vs. Average

Our hierarchical Bayesian model was the best predictor of individual participants’ behavior, followed by the No-Transfer model, an ablation of the full model that assumes that people treat each causal system as an independent learning problem. The No-Transfer model also had the best predictions in a group-level, averaged analysis that assumes all participants have a common strategy. This result is likely due to the No-Transfer model bringing a more diffuse prior to the transfer task, which we discuss next.

Since the No-Transfer model removes the hypothesis that people transfer what they learned from the previous training tasks, it starts anew with a prior that has not been tuned to any previous learning. This prior is more diffuse and thus assigns moderate probabilities to a large set of hypotheses, making it fairly consistent with several strategies that are informative about any of these hypotheses. For example, these strategies could include our full model’s strategies, which are informative under transferred overhypotheses, or less cognitively demanding strategies, where even random strategies can provide information about *some* set of hypotheses. Thus, the No-Transfer model would be, on average, a good predictor for all of these different strategies. However, the average model does not explain how people can also deviate from the average and behave differently from each other. Moreover, it is often the case that the average model may not capture the behavior of any individual (e.g., Hayes, 1953; Estes, 1956; Ashby et al., 1994; Heathcote et al., 2000), and this phenomenon is also pertinent to average models of causal learning (Johnston et al., 2021).

An inspection of our data reveals several qualitatively distinct strategies (Fig. 9) that were unlikely to be captured by an average model, which is consistent with our individual differences analysis showing different participants adopting strategies consistent with different models. This diversity in strategies, including some that may result in lower judgment accuracy or less informative interventions while imposing lower demands on memory, time, or attention, is consistent with both variability in participants’ prior beliefs and a “resource-rational” view of inductive learning (Griffiths et al., 2015; Lieder & Griffiths, 2017). For example, if a participant is not motivated to perform well in the task itself but is rather only concerned with completing the task and receiving the base compensation (independent of task performance), they might choose a cost-efficient strategy that is more consistent with a random model, or an attenuated hypothesis space. Under this view that individuals vary in their implicit cost/performance trade-offs, greater performance incentives (e.g., bonuses to our crowdsourced participants) may increase the proportion of participants who are best-predicted by our full hierarchical Bayesian model.

The No-Transfer ablation model also performed well in our individual differences analysis and captured a sizable, second-largest group of individuals. These individuals may have had sufficiently strong prior beliefs favoring deterministic and disjunctive relationships (Lucas & Griffiths, 2010; Lu et al., 2008; Mayrhofer & Waldmann, 2016; Schulz & Sommerville, 2006) that they treated the conjunctive and 3-conjunctive training conditions as outliers or special cases, unlikely to generalize to new machines. Consequently, they may have resorted to the same deterministic- and disjunctive-favoring prior in the next task, as is consistent with the No-Transfer model. Such behavior is also consistent with how people generalize causal laws across several tasks (Zhao et al., 2022). In other words, they may not have taken the high degree of superficial similarity between our experiment’s training and transfer tasks to be a strong indicator that the underlying causal relationships would have the same form, contra our full model.

Although the No-Transfer participants were not captured by our full model, we note that their behavior was still consistent with the other two hypotheses that were retained in the No-Transfer model. Like our full model, the No-Transfer model ascribes to participants (1) rich overhypotheses about the functional form of relationships and commits to the idea that (2) participants seek to learn about these functional forms. Both of these hypotheses go beyond past models of active causal learning and were required to predict the majority (73.6%) of participants’ interventions (combining both HBM and No-Transfer participants in Fig. 8). Ultimately, the largest group of participants was best-predicted by our full model, suggesting that all three modeling hypotheses, including the transfer hypothesis, were required to capture the dominant strategy that people employ in actively learning to learn causal relationships.

### Beyond the Sigmoid Space

Following Lucas and Griffiths (2010), we chose our space of functional forms by assuming (1) causes are interchangeable, without any distinct subtypes of causes; (2) as the number of active causes grows, so does the probability of the target effect; (3) causes may or may not be individually sufficient to generate the effect, and (4) relationships can (but need not) be deterministic. These assumptions are captured by letting the probability of the effect (blicket machine activation) be a sigmoid function of the number of causes (blickets). It captures several qualitatively distinct relationship types, including all of the forms in our experimental manipulations, while being computationally efficient, interpretable, and having only two parameters.

While it is appealing to consider a much wider space of forms, this would present several challenges for our experimental design and modeling. First, even just studying the transfer effects of disjunctive and conjunctive forms involves an expensive experimental design with several between-subjects conditions, as has been shown in past experiments of causal overhypotheses (e.g., Lucas & Griffiths, 2010; Lucas et al., 2014; Kosoy et al., 2022) and in our Experiment 1. Therefore, we limit our experiments to manipulate only a few new forms (Experiment 2) while retaining the standard disjunctive and conjunctive forms as points of comparison (Experiments 1 and 2), creating 14 total between-subjects conditions. Second, it is not clear which forms beyond the sigmoid space would be appropriate to represent in our model. We chose the sigmoid space in light of its success in explaining human behavior in similar settings (Lucas & Griffiths, 2010; Lucas et al., 2014) and its match to our experiment’s demand characteristics. Our experiment’s cover story follows the blicket detector paradigm, which is commonly used to study causal overhypotheses (e.g., Lucas & Griffiths, 2010; Griffiths et al., 2011; Lucas et al., 2014; Sim & Xu, 2017; Kosoy et al., 2022). This cover story aims to set up expectations that causal relationships are simple and discoverable, and that these relationships have the property where blickets are interchangeable and generative causes of the blicket machine’s binary activation (see the instructions for participants in Appendix B, and see https://osf.io/vk9yd, where we ran model simulations to ensure relationships are discoverable in Experiment 2). These demand characteristics are well-matched to the simplicity and assumptions of the sigmoid space.

In more general settings, however, people may learn and transfer a broader space of forms than can be encompassed by the sigmoid space, such as forms that combine preventative and continuous causes (Yuille & Lu, 2007; Lu et al., 2016). Therefore, an exciting future direction is to go beyond parametric functional forms, and consider arbitrarily expressive belief spaces. For example, grammar-based and program induction methods offer suggestions about how people can dynamically and compositionally expand their belief space with an infinite set of possible concepts (e.g., Goodman et al., 2008; Lake et al., 2015; Goodman et al., 2015; Piantadosi et al., 2016).

### Reinforcement Learning

An alternative family of models for active learning originates in the reinforcement learning literature. Models based on reinforcement learning have successfully explained cognitive phenomena (e.g., Dayan & Niv, 2008) and provided computational solutions to complex active learning problems (e.g., Vinyals et al., 2019; Wurman et al., 2022). These models typically require thousands of actions and task repetitions where a human only requires a few, but recent advances have begun to leverage abstract knowledge that can be shared between tasks and thus allow a reinforcement learning model to learn more efficiently in new tasks (Hospedales et al., 2022; Tomov et al., 2021; Eckstein & Collins, 2020; Zhang et al., 2021). However, it is still a challenge for these models to incorporate certain kinds of abstract knowledge and inductive biases that align with human behavior, especially human causal learning. For example, modern reinforcement learning agents have difficulty learning abstract causal knowledge in the Alchemy benchmark (Wang et al., 2021), which was designed with inspiration from studies of human learning. Furthermore, with regard to blicket tasks that are similar to our experiments, it remains an open direction how current reinforcement learning agents can explore like children (Kosoy et al., 2022), who consider rich priors for causal overhypotheses that are much like the overhypotheses studied in our current work. This is not to say the reinforcement learning approach would be ineffective for modeling how humans actively learn to learn, but there are open questions about how this approach can achieve the same learning efficiency and inductive biases as humans. Thus, in our work, we have chosen a hierarchical Bayesian model that can learn from the same number of interventions as each participant, and can straightforwardly represent and learn about overhypotheses that are supported by studies of human behavior (Lucas & Griffiths, 2010; Lucas et al., 2014).

## Conclusion

Overall, we explored the question of how humans choose actions to facilitate long-term learning and make their future actions more efficient. In other words, how do people actively learn to learn? We focused on the domain of active causal learning, where past models (e.g., Steyvers et al., 2003; Bramley et al., 2015; Coenen et al., 2015) have made an important simplifying assumption by predicting that interventions are only informative about the causal relationship at hand, which would not explain how people can acquire general knowledge that is useful outside of their current situation and exploit such knowledge to choose more efficient future interventions. We proposed and found evidence for a hierarchical Bayesian model, which differs from these earlier models in one key way: It posits that people not only seek information about the causal relationship at hand, but also balance this with the goal of learning and transferring *overhypotheses* (Goodman, 1955; Kemp et al., 2007) that are useful for future causal learning problems.

Our approach can generalize beyond causal learning to active learning in any setting where there is an opportunity for learning about the abstract properties of the task, i.e., for updating and exploiting overhypotheses. Examples range from graph structure learning (Mansinghka et al., 2006) to the optimal stopping problem (Lee, 2006) to category learning (Kemp et al., 2007). Thus, accounting for overhypotheses may provide a foundation for working toward a better understanding of a wide range of domains where humans actively learn to learn.

## Supplementary Information

Below is the link to the electronic supplementary material.

Supplementary file 1 (pdf 334 KB)

## Data Availability

Experimental data and online experiment materials will be made publicly available upon publication. Experiment 1 analysis code is here. Experiment 2 modeling code is here and here.

## Footnotes

1 This is typical of real-world LEDs, which, unlike incandescent bulbs tend to illuminate only when a threshold voltage is exceeded, which can require a circuit with 2–3 standard AA batteries in series. For simplicity, we restrict our examples to series circuits.

2 The computational complexity of computing expected information gain grows more than exponentially in the number of blocks, as the space of causal structure hypotheses and possible interventions are both power sets of the set of prospective causes (i.e., the blocks) and contribute multiplicatively to the total computational cost. The reduction in the hypothesis and action space allowed us to run all models on a compute cluster in 1 week rather than an estimated 8 weeks.

3 This figure displays weights fitted on normalized values of form and structure EIG. In Appendix A, we reproduce the central result of this figure using unnormalized values.
