# Supplementary material for: Actively Learning to Learn Causal Relationships
Source: Comput Brain Behav. 2024 Jan 5;7(1):80–105. doi: 10.1007/s42113-023-00195-0 (PMC13292816; doi:10.1007/s42113-023-00195-0)
Supplement: Supplementary file 1 — (pdf 334 KB) [file 42113_2023_195_MOESM1_ESM.pdf]

Appendix A Supplementary Results

A.1 Experiment 1

A.1.1 Causal judgments

Further results for blicket identification judgments

We expected causal judgment accuracy to be predicted by the match between the transfer and training functional forms, training length, and their interaction, considering the transfer task's functional form as a covariate. We used these variables to fit a logistic regression model to predict the per-participant accuracy percentage in the transfer task's blicket identification questions (binomial with 9 trials). We confirmed a significant main effect of the match of functional form ($z = 2.62, p = .009$; filtered: $z_f = 3.25, p_f = .001$). The transfer task's functional form also had a significant main effect ($z = 3.99, p < .001$; filtered: $z_f = 4.78, p_f < .001$), which was consistent with past results suggesting that people find disjunctive forms easier to learn (Lucas and Griffiths, 2010). The length of training and its interaction with the match between training and transfer forms were not significant predictors, which may be attributable to a weaker match effect in specifically the conjunctive transfer conditions, as shown by our t-tests in Section 4.2.

Activation prediction judgments

Aside from blicket identification judgments, we also considered another type of causal judgment in the transfer task: 7 predictions about whether or not a combination of blocks would activate the blicket machine (see Section B for more details). Like for the blicket identification judgments, we expected the activation prediction accuracy to be predicted by the match between the transfer and training functional forms, training length, and their interaction, considering the transfer task's functional form as a covariate. We used these variables to fit a logistic regression model to predict the per-participant accuracy percentage in the transfer task activation prediction questions (binomial with 7 trials). The main effect of the match of functional form was not significant in the full data ($z = 1.24, p = .215$), but was significant for the filtered participants who were more engaged with the transfer task ($z_f = 2.53, p_f = .012$). The transfer task's functional form had a significant main effect ($z = 3.67, p < .001$; filtered: $z_f = 4.23, p_f < .001$), which was consistent with past results suggesting that people find disjunctive forms easier to learn (Lucas and Griffiths, 2010). Surprisingly, the length of training and its interaction with the match between training and transfer forms were not significant predictors.

We also used Welch t-tests (two-tailed) to investigate the specific effects of match between pairs of conditions (visualized in Fig. A1b), expecting causal judgment accuracies to improve from mismatched to matched conditions. In the disjunctive transfer conditions, the comparisons were consistent with our expectations: Mean activation prediction accuracy improved significantly from mismatched to matched conditions with long training, $t(50.00) = -3.04, p =$

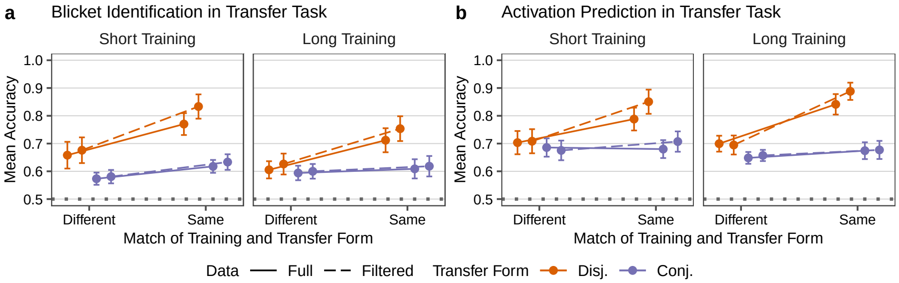

Fig. A1: Experiment 1: Questionnaire performance in the transfer task, grouped by the transfer functional form ("Disj." for Disjunctive, or "Conj." for Conjunctive), its match with the training form (Same or Different), and training length (Long or Short). Chance (.5) accuracy is shown with a dotted gray line. Error bars in either direction denote the magnitude of the standard error. Mean participant accuracies for **a** blicket identification and **b** activation prediction are calculated separately for the full and filtered data.

.004 (filtered: $t_f(42.37) = -4.18, p_f < .001$). The short training improvement was not significant in the full data ($t(52.70) = -1.45, p = .154$), but was significant in the filtered data ($t_f(43.89) = -2.31, p_f = .025$). In the conjunctive transfer conditions, however, the difference between matched (conjunctive training) and mismatched (disjunctive training) accuracies was non-significant. This weaker match effect might have accounted for the non-significant interaction effect between match and training length in our logistic regression model. We suspected this weaker effect was due to the conjunctive transfer task being too difficult to learn, regardless of training match and length. This suspicion was supported by the blicket identification results in our next experiment, where we lowered the difficulty of the conjunctive transfer task and found a significant improvement from mismatched to matched conditions (see Section A.2).

A.1.2 First intervention

Further results for blicket identification judgments

To further test whether the first intervention in the transfer task was informative under the training form, we used a linear model to predict the number of blocks in the first intervention, where the predictors were the training form, the training length, and their interaction. There was a significant interaction effect ($t(205) = -3.59, p < .001$; filtered: $t_f(177) = -3.46, p_f < .001$) and significant main effect of training length ($t(205) = 3.38, p < .001$; filtered: $t_f(177) = 3.66, p_f < .001$). The non-significant main effect of the training form ($p \geq .322$ for both the full and filtered data) may be attributable to weaker effects in the short conditions—see Fig. 5.

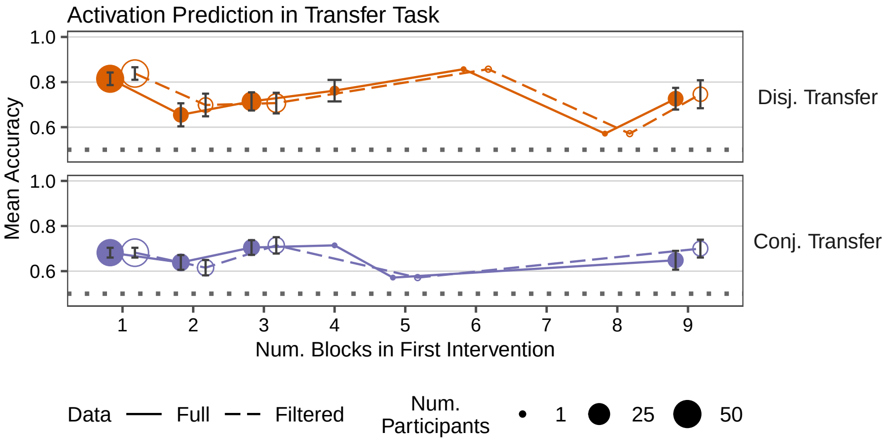

Fig. A2: Experiment 1: Mean participant accuracies for activation prediction questions in the transfer task. This is grouped by the number of blocks in the first intervention and the transfer form. The mean is calculated separately for the full (solid lines) and filtered (dashed lines) data. Error bars in either direction denote the magnitude of the standard error but are omitted for points with a single participant, where the standard error is ill-defined. Chance (.5) accuracy is shown with a dotted gray line.

Activation prediction judgments

To understand when the first intervention would be efficient for learning in the transfer task, we fitted a (binomial) logistic regression model to predict activation prediction accuracy (7 trials). The predictors included the number of blocks in the first intervention, the functional form of the transfer task, and their interaction. There was a significant main effect of the transfer form ($z = 3.38, p < .001$; filtered: $z_f = 4.29, p_f < .001$), but no other significant effects (all $p \geq .078$). Our figures suggest that even though participants were able to identify a larger subset of blickets with efficient interventions (see Fig 6 in the main text), this partial knowledge was not sufficient to make more accurate activation predictions (Fig. A2), which had a larger coverage over blickets and their combinations with other blocks.

A.2 Addressing Experiment 1's Non-Significant Results

In Experiment 1, the conjunctive transfer task conditions had a non-significant difference in blicket judgments between matched (conjunctive) and mismatched (disjunctive) training for both long (2 tasks) and short (1 task) training lengths. We had expected a strong effect of matched vs. mismatched training, especially in the longer training conditions that gave additional opportunities to learn about the matched or mismatched form. Instead, we found a non-significant trend. One possible explanation would be that participants were not learning conjunctive overhypotheses through training, and thus,

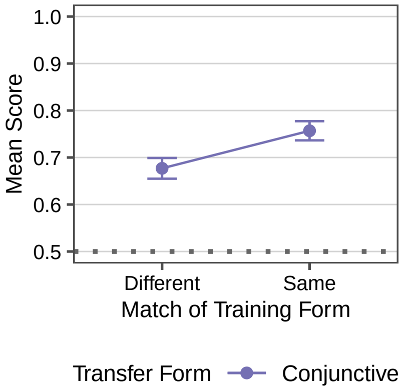

Fig. A3: Experiment 2: Mean blicket rating score in the conjunctive transfer task. The plotted mean scores are grouped by whether the training functional form was the same (deterministic conjunctive condition) or different (deterministic disjunctive condition). A participant's blicket rating (0-10) is scored as $1 - \frac{|\text{participant rating} - \text{true rating}|}{10}$, where the true rating is 10 for blicket blocks and 0 for non-blicket blocks. These scores are averaged over participants and each of their 6 ratings (one for each block) in the transfer task. The chance level score (.5) is shown with a dotted gray line and error bars in either direction denote the magnitude of the standard error. This plot shows participants' scores in the conjunctive transfer task improved from mismatched to matched training.

participants with matched conjunctive training were performing no better than those with mismatched disjunctive training. However, this explanation seems unlikely and it is possible participants were fatigued or frustrated due to the difficulty of Experiment 1's conjunctive transfer task, which involved finding 4 blickets among 9 blocks (which can be intervened on in $2^9 = 512$ ways) within 45 seconds. For example, a simple and reasonable strategy under a conjunctive form would be to intervene on only the 36 possible *pairs* of blocks, but even this was not possible under the short time limit. In contrast, the same time limit allowed participants to successfully find blickets in the easier disjunctive variant of the transfer task by testing all 9 singleton blocks. Indeed, performance was lower in the conjunctive transfer task conditions than in the disjunctive transfer task conditions ($p < .001$ for both blicket classification and activation prediction questions in the full and filtered data).

To address the difficulty of Experiment 1's conjunctive transfer task, we designed an easier version for Experiment 2, asking participants to find 3 blickets among 6 blocks (which can be intervened on in $2^6 = 64$ ways) with a fixed intervention number of 20. In this easier conjunctive transfer task, we now found a significant improvement in mean blicket judgments from mismatched to matched training (two-tailed Welch t-test: $t(85.856) = -2.17, p = .033$; comparison is visualized in Fig. A3), where the mismatched and matched training correspond to the deterministic disjunctive and deterministic conjunctive

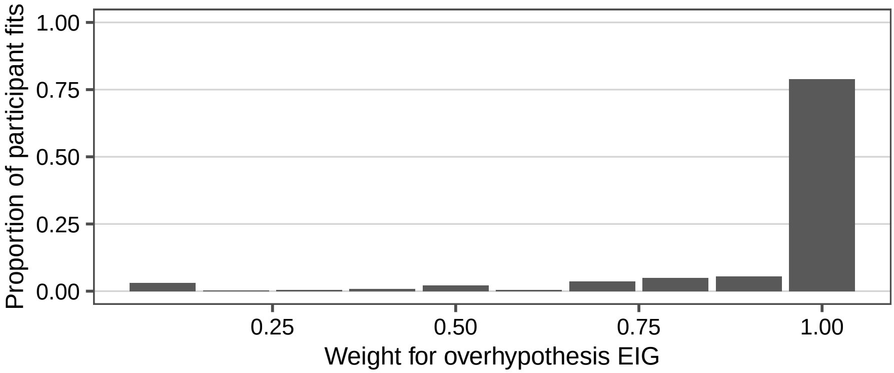

Fig. A4: Weighting of overhypothesis vs. causal structure EIG *with unnormalized EIG* (Experiment 2; HBM participants). This plot is equivalent to Fig. 10 without EIG normalization.

training conditions in Experiment 2. Thus, Experiment 2's results address Experiment 1's non-significant differences in blicket judgments.

Experiment 2's conjunctive transfer task had a few more differences compared with Experiment 1's. Experiment 2 asked participants to rate blickets on a 0-10 scale instead of asking them to classify them on a binary scale (blicket or not). The blicket rating scale follows Lucas and Griffiths's (2010) measure of disjunctive versus conjunctive training effects in a passive learning setting and allows us to measure these effects more precisely in our active learning setting as well. Experiment 2 also did not vary the training length like Experiment 1, but instead used only a short (1 task) training in all its conditions. However, since we already see a significant effect in Experiment 2's short training, we expect that this effect would remain or be larger with longer training.

A.3 Experiment 2

A.3.1 Weighting overhypotheses vs. causal structures

In the main results, we analyzed the distribution of fitted overhypothesis weights for individuals best-predicted by our HBM (Fig. 10). These weights were fitted after normalizing overhypothesis EIGs and structure EIGs to be in the same [0,1] range (see Section 3.3). This normalization accounts for softmax's sensitivity to different ranges of overhypothesis and structure EIGs when computing predictive likelihoods. Here we plotted the distribution of fitted weights for unnormalized EIG values (Fig. A4) and verified that the general shape of the distribution is unchanged, i.e., there is still a pronounced skew toward higher overhypothesis weights. The main difference is that a more extreme proportion of fits have an overhypothesis weight of 1, which is likely due to softmax's scale sensitivity.

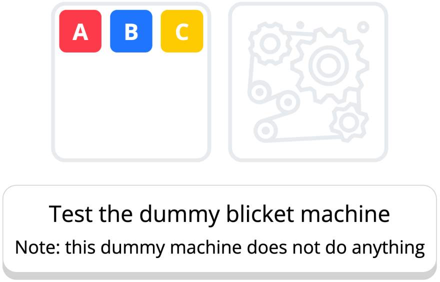

Fig. B5: Dummy blicket machine and blocks used in the instructions for participants. To prevent biasing participants toward a particular functional form, we communicated that the machine "does not do anything" in response to the blocks. Rather, this setup was used as an interactive tool for participants to learn about clicking/-moving blocks and pressing a button to test the machine.

Appendix B Supplementary Methods

B.1 Instructions for participants

Below, we paste the main instructions that we gave participants in Experiment 2, which are the same as the instructions for Experiment 1 except for minor word edits.

The Blicket Game

The blicket game involves blocks with different letters and colors. Some blocks have special properties that make them **blickets** and your goal is to identify these blickets with the help of a **blicket machine**. *Only* the blicket machine can help us identify blickets. A block's color and letter don't tell us anything about whether it is a blicket.

Here's an example of some blocks (A, B, C) and a dummy blicket machine (square with cogs):

[see Fig. B5]

Try clicking on the blocks (A, B and C) above! This allows us to move any number of blocks on or off the blicket machine. Press the test button to see a dummy response from the blicket machine.

In the **real blicket game**, the test button will show how the blicket machine responds to different combinations of blocks: the machine can either "activate" with a green color, or do nothing. It doesn't matter where blocks are placed on the machine.

B.2 Experiment 1

In addition to blicket identification questions, the questionnaire after each task also included binary predictions of whether the blicket machine would activate in the presence of different combinations of blocks ("Will the blicket machine activate (light up with a green color)?"). There were seven different predictions about seven different combinations of blocks, including combinations with zero, one and two blickets along with other non-blicket blocks (where the number and identities of blickets and non-blickets were unknown to participants), as well as one combination with all blocks in the task. For example, consider a transfer task with the nine blocks {J, K*, L*, M, N, O, P, Q*, R*} (where blickets are marked with an asterisk for the sake of this example). The seven combinations (subject to randomization of the exact blickets and non-blickets) could then be {N, O, Q*}, {J, M, R*} (one blicket); {P, Q*, R*}, {K*, L*, O} (two blickets); {J, N, O}, {M, N, O, P} (zero blickets); and finally one combination containing all nine blocks.
